# CD73 promotes non–small cell lung cancer metastasis by regulating Axl signaling independent of GAS6

**DOI:** 10.1073/pnas.2404709121

**Published:** 2024-10-18

**Authors:** Jianjie Zhu, Wenwen Du, Yuanyuan Zeng, Ting Liu, Jianjun Li, Anqi Wang, Yue Li, Weijie Zhang, Jian-an Huang, Zeyi Liu

**Affiliations:** ^a^Department of Pulmonary and Critical Care Medicine, The First Affiliated Hospital of Soochow University, Suzhou 215006, China; ^b^Institute of Respiratory Diseases, Soochow University, Suzhou 215006, China; ^c^Suzhou Key Laboratory for Respiratory Diseases, Suzhou 215006, China

**Keywords:** CD73, Axl, tumor metastasis, tumor subtype, non–small cell lung cancer

## Abstract

As catabolic enzyme, CD73 dephosphorylates adenosine monophosphate (AMP) and can also regulate tumor cell proliferation and metastasis. To date, very few studies have explored the role of CD73 in mediating non–small cell lung cancer (NSCLC) metastasis, and the underlying transducing signal has not been elucidated. In the present study, we demonstrated that the CD73/Axl axis could regulate Smad3-induced epithelial-to-mesenchymal transition (EMT) to promote NSCLC metastasis. Mechanically, CD73 can be secreted via the Golgi apparatus transport pathway. Then secreted CD73 may activate AXl by directly bind with site R55 located in Axl extracellular domain independently of GAS6. In addition, we proved that CD73 can stabilize Axl expression via inhibiting CBLB expression. We also identified the distinct function of CD73 activity in adenocarcinoma and squamous cell carcinoma. Our findings indicated a role of CD73 in mediating NSCLC metastasis and propose it as a therapeutic target for NSCLC.

## Significance

Cancer metastasis is the dominant cause of mortality in non–small cell lung cancer (NSCLC) patients. Therefore, identification of metastasis-promoting molecules is important for cancer management and research. We demonstrate that CD73 was highly expressed in NSCLC tissues and cell lines, and overexpression of CD73 can promote NSCLC cell metastasis via the Axl/Smad3 signaling pathway. Moreover, we found that secreted CD73 can directly bind with site R55 located in Axl extracellular domain to serve as a ligand for Axl to induce its activation. Our findings indicated a role of CD73 in mediating NSCLC metastasis, especially for lung squamous cell cancer patients with high CD73 expression, combined targeting of AXL may provide the greatest benefit to patients.

CD73 is a glycosyl-phosphatidylinositol (GPI)-anchored membrane glycoprotein and plays a significant role in tumor progression (1). As a catabolic enzyme in purine metabolism, CD73 can dephosphorylate adenosine monophosphate (AMP) into adenosine, which interacts with all types of G-protein-coupled cell surface receptors (A1, A2A, A2B, and A3) to regulate tumor behavior (2, 3). The immunosuppressive function of adenosine can robustly suppress proliferation and regulate function of various immune cells, including T cells, regulatory T cells (Tregs), and myeloid-derived cells (MDSCs). This can generate an immunosuppressive microenvironment and shield cancer cells from host immunity, leading to tumor immune escape (4, 5). Evaluation of small-molecule CD73 inhibitors and anti-CD73 monoclonal antibodies in preclinical studies has highlighted CD73 as a potential checkpoint for cancer therapy (6, 7). In addition to immunosuppressive functions, emerging evidence has demonstrated that CD73 can enhance cancer cell proliferation and migration, and participate in chemotherapy resistance (8).

The role of CD73 in cancer metastasis has been widely demonstrated in solid tumors (9). In gastric cancer, CD73 promotes tumor metastasis by modulating RICS/RhoA signaling and epithelial to mesenchymal transition (EMT) (10). In amoeboid pancreatic cancer, CD73 activates RhoA-ROCK-Myosin II-driven cell invasion and metastasis (11). Limited studies have reported the role of CD73 in lung cancer metastasis. In lung adenocarcinoma, CD73 promotes the proliferation and metastasis through the EGFR/AKT/mTOR pathway (12). Another study showed that knockdown of CD73 can decrease NSCLC cell viability and metastasis and enhance chemosensitivity to cisplatin by down-regulating c-Myc, MMP-9, and ROCK expression (13). Our previous study showed that CD73 is frequently overexpressed in NSCLC tissues and cell lines, CD73 knockdown also inhibits cell migration in vitro (14). However, the mechanism by which CD73 transduces signals during tumor metastasis is not well understood.

Under normal conditions, CD73 functions as a glycosylphosphatidylinositol (GPI)-anchored protein; however, it can be released from the cell membrane via cleavage by metalloproteinases or shed by cell-associated phospholipases (15). A highly glycosylated isoform of soluble CD73 was detected in the plasma of cervical cancer patients and correlated with the disease progression (16). In metastatic colorectal cancer patients receiving chemotherapy with bevacizumab or cetuximab, high plasma level of CD73 was identified as a prognostic factor of worse progression-free survival (PFS) and overall survival (OS) (17). In addition, exosome-derived CD73 is a prognostic factor in patients with metastatic melanoma, which suppresses lymphocyte function, and is associated with the response to anti-PD-1 therapy (18). CD73-positive extracellular vesicles promote glioblastoma immunosuppression by inhibiting clonal expansion of T cells (19). However, the expression and function of soluble CD73 in lung cancer remain unknown.

Axl is a member of the TAM family of receptors tyrosine kinases (RTKs) and is implicated in tumor progression, cell plasticity, and metastasis in pancreatic cancer (20). In hepatocellular carcinoma, Axl/14-3-3ζ signaling is central to TGF-β-mediated cell progression and is a promising target for HCC therapy (21). The prometastatic role of Axl in NSCLC is also discussed. In the literature, Axl expression varies from 33 to 93.2% in lung cancer tissues, implying poor prognosis and disease progression (22). Dual inhibition of MEK and Axl targets tumor cell heterogeneity and prevents EMT-mediated cell growth and metastasis in NSCLC (23). The TAZ-Axl-ABL2 feed-forward signaling axis can promote lung adenocarcinoma brain metastasis (24). However, how Axl becomes activated in NSCLC remains unclear. Conventional activation of Axl is mediated by its ligands GAS6 or PROS1. Axl can also bind to other members of the TAM family to form transduce downstream signaling pathways (25). Therefore, whether soluble CD73 can interact with Axl and induce its activation to contribute to NSCLC metastasis remains to be explored.

This study shows the molecular collaboration between CD73 and Axl signaling in NSCLC cell migration. Analysis of primary NSCLC samples and cell lines showed a positive correlation between CD73 and Axl expression. We demonstrated that CD73 can be secreted in soluble form and serve as a ligand for Axl to induce its activation and thus promote cell metastasis. CD73 can stabilize Axl expression via inhibiting E3 ubiquitin ligases CBLB expression. More importantly, we pointed out the distinct role of CD73 enzyme activity of NSCLC subtypes in regulating cell metastasis. This molecular mechanism further suggests that CD73 is a target to combat NSCLC progression.

## Results

### CD73 Augmented Cell Migration and Invasion in NSCLC.

**Fig. 1.**
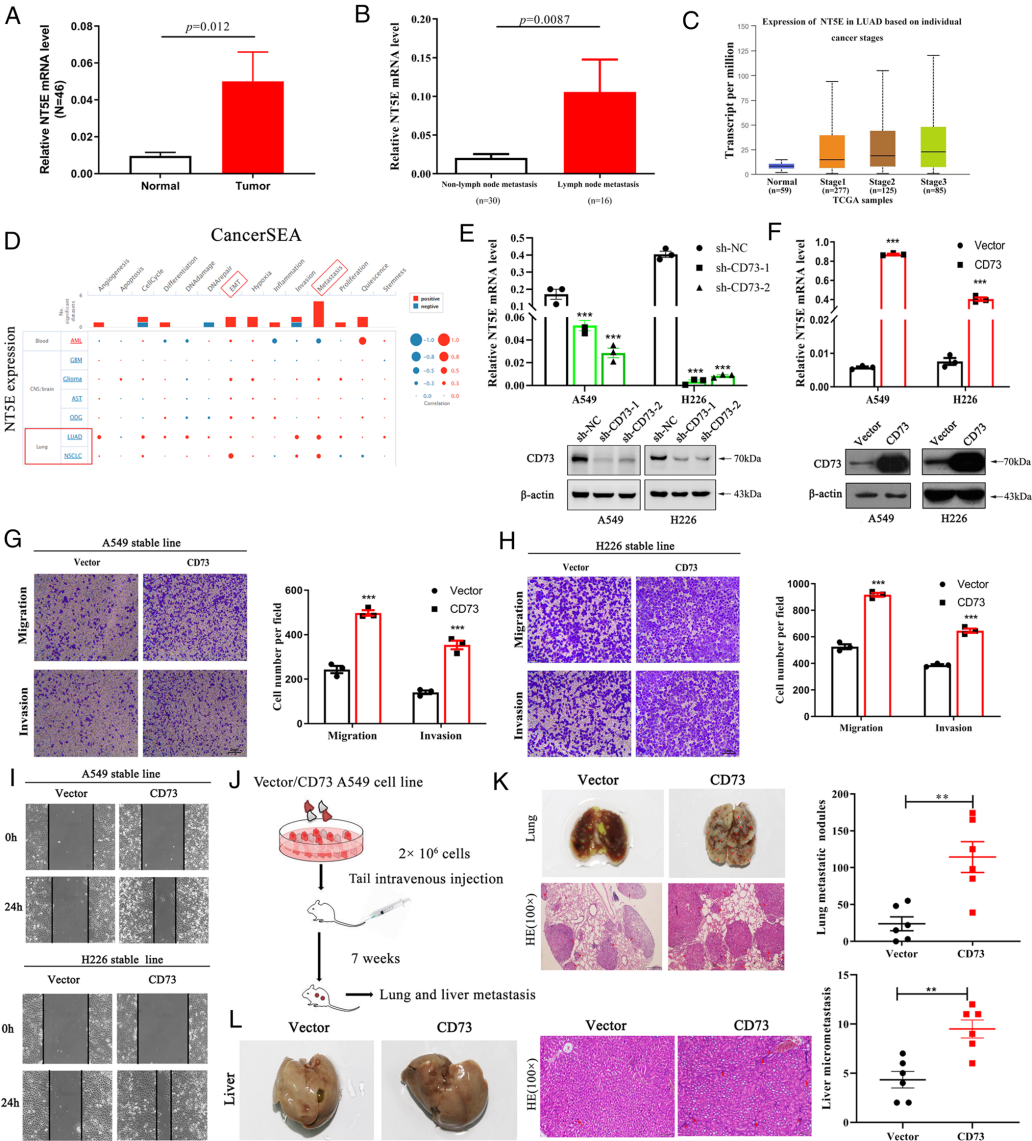
CD73 regulates NSCLC cell metastasis. (*A*) NT5E mRNA expression in 46 paired lung cancer tissues. (*B*) NT5E mRNA expression in metastatic lymph node tissues. (*C*) NT5E expression in LUAD cancer tissues under different TNM stages based on TCGA database. (*D*) The association between NT5E expression and EMT/ metastasis via CancerSEA database. (*E* and F*)* NT5E mRNA and protein expression levels after stable knockdown and overexpression in A549 and H226 cell lines via qRT-PCR and western blot assays. (*G* and *H*) Transwell assay of A549 and H226 cells after CD73 overexpression. (*I*) Wound healing assay of A549 and H226 cells after CD73 overexpression. (*J*) Pattern diagram of the tumor metastatic model. (*K* and *L*) Representative images of surface pulmonary metastasis nodules and liver metastasis after CD73 overexpression in the murine model and relative quantitative numbers of pulmonary and liver metastasis nodules.

First, we found that CD73 expression is higher in 46 paired lung cancer tissues when compared to normal lung tissues (Fig. 1*A*). Moreover, increased CD73 expression was demonstrated in metastatic lymph node tissues (Fig. 1*B*). Data extracted from TCGA database showed that CD73 expression is elevated along with advanced TNM stages (Fig. 1*C*). Based on single-cell sequencing data obtained from the CancerSEA database also showed that NT5E expression level was positively correlated with EMT and metastasis in lung adenocarcinoma and NSCLC tissues (Fig. 1*D*). All above data indicated that CD73 might participate in NSCLC metastasis.

To test the role of CD73 in mediating cell metastasis, we constructed CD73 stable knockdown and overexpression cell lines, and the expression level of CD73 was confirmed at both the mRNA and protein levels (Fig. 1 *E* and *F*). Next, we evaluated the role of CD73 in mediating the migration and invasion of NSCLC cells. Transwell assays indicated that CD73 overexpression promoted cell migration and invasion (Fig. 1 *G* and *H*), and knockdown of CD73 led to the opposite phenomena (*SI Appendix*, Fig. S1*A*). The wound-healing assay also demonstrated that CD73 overexpression resulted in a marked increase in wound closure at 24 h (Fig. 1*I*). In contrast, cells with stable CD73 knockdown had a lower ability to migrate (*SI Appendix*, Fig. S1*B*). To further assess the effect of CD73 on NSCLC metastasis in vivo, we injected the CD73-overexpressed A549 cells and control cells into the tail veins of nude mice (Fig. 1*J*). As shown in Fig. 1 *K* and *L* and *SI Appendix*, Fig. S1*C*, more surface pulmonary and liver metastasis nodules were observed in the CD73 overexpression group. H&E staining was performed to histologically detect changes in lung and liver tissues.

### CD73 Interacted with and Induced Axl Activation in NSCLC Tissues and Cell Lines.

**Fig. 2.**
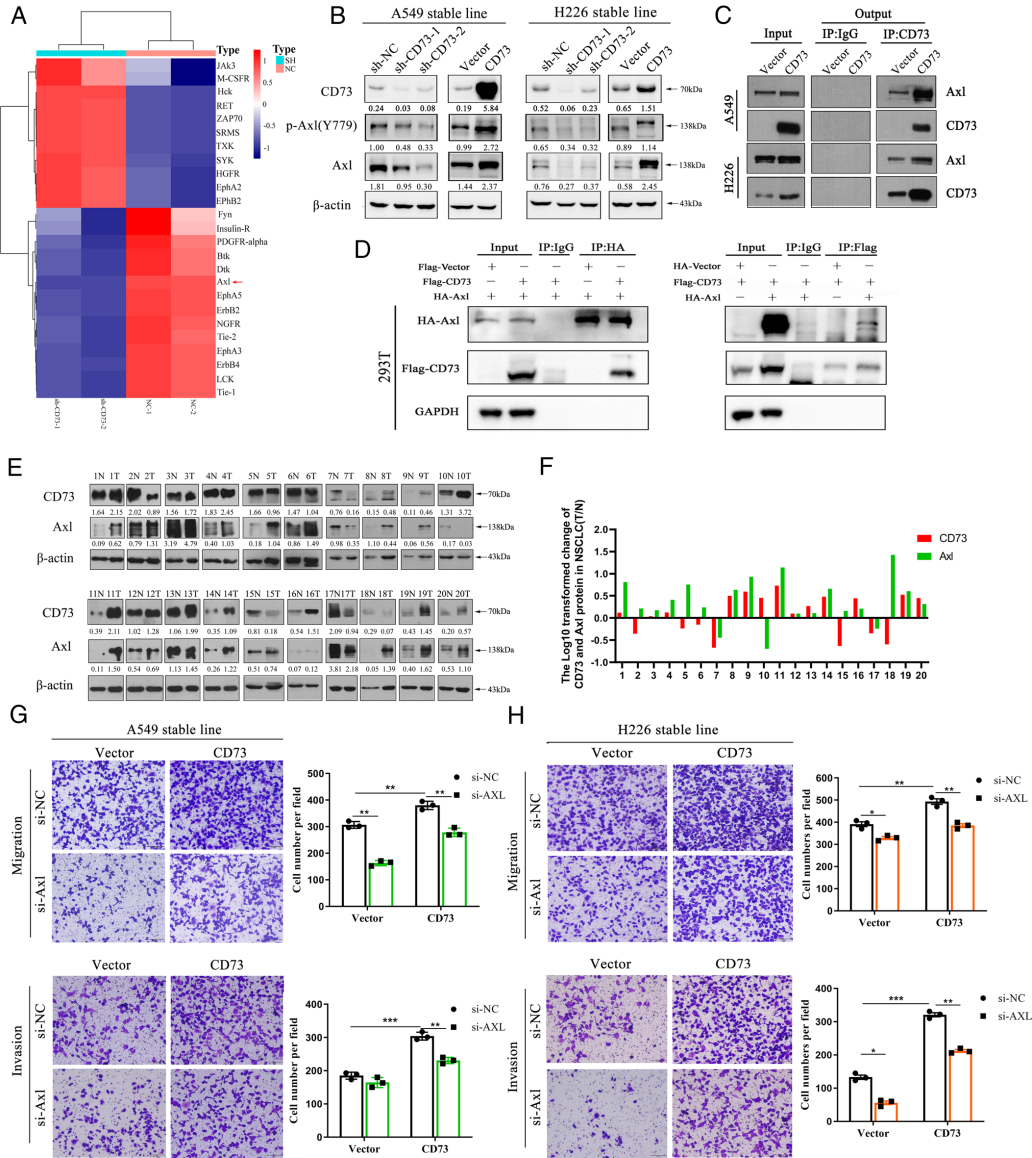
CD73 interacts with and induces Axl activation in NSCLC tissues and cell lines. (*A*) Tyrosine receptor kinase protein array of associated molecules after CD73 knockdown. (*B*) Western blot assay of Axl and p-Axl expression after CD73 knockdown and overexpression. (*C* and *D*) Co-IP assay was performed to assess the interaction between CD73 and Axl. (*E*) Western blot assay of CD73 and Axl protein levels in 20 paired NSCLC tissues and the corresponding adjacent tissues. Correlation between CD73 and Axl protein levels in 20 paired NSCLC tissues. (*F*) Relative quantification of CD73 and Axl protein levels in 20 paired NSCLC tissues. (*G* and *H*) Transwell assay in CD73-overexpressed cells and control cells after Axl knockdown via siRNA.

Human RTK Phosphorylation Antibody Array revealed that Axl phosphorylation was down-regulated in CD73-knockdown cells compared to control cells (Fig. 2*A* and *SI Appendix*, Fig. S2*A*). CancerSEA database showed that Axl expression levels were positively correlated with EMT and metastasis in lung adenocarcinoma tissues (*SI Appendix*, Fig. S2*B*). The GeneMANIA database indicated that NT5E expression levels correlated with Axl and GAS6 expression (*SI Appendix*, Fig. S2*C*). We then validated the results in CD73-stable knockdown and overexpressed cell lines. We found that CD73 knockdown decreased the phosphorylation level of Axl and total Axl expression, while CD73 overexpression up-regulated total Axl and p-Axl (Fig. 2*B*). Co-IP assays showed that CD73 interacted with Axl in both A549 and H226 cell lines (Fig. 2*C*). We further coexpressed HA-tagged Axl and Flag-tagged CD73 in HEK293T cells to verify the above findings. Axl was detected in CD73 immunoprecipitates, indicating that CD73 interacts with Axl (Fig. 2*D*). We also confirmed these findings in patient samples. A summary of patient characteristics and correlation coefficients with CD73 expression is listed in *SI Appendix*, Table S1 and S2. Among the 20 randomly selected paired NSCLC tissues, 14 pairs were positively correlated of CD73 and Axl expression and two pairs were negatively correlated (Fig. 2 *E* and *F*). Finally, transwell assay showed that Axl knockdown diminished CD73-induced increased migratory and invasive abilities in both A549 and H226 overexpressing cell lines (Fig. 2 *G* and *H*). Taken together, the above results indicate that CD73 promotes non–small cell lung cancer (NSCLC) metastasis through Axl signaling.

### GAS6 was not Essential for CD73-induced Axl Activation in Mediating NSCLC Cell Metastasis.

Next, we investigated how CD73 activates Axl signaling in NSCLC cell lines. GAS6 is a well-recognized ligand for Axl receptor activation. Therefore, we measured GAS6 mRNA expression in CD73 stable cell lines. We found that GAS6 mRNA expression levels remained unchanged in both CD73 knockdown and overexpressed cell lines (*SI Appendix*, Fig. S3 *A* and *B*). ELISA data also showed that CD73 overexpression failed to increase the secreted GAS6 levels in the supernatant (*SI Appendix*, Fig. S3*C*). In addition, we knocked down GAS6 expression in parental cells and CD73 overexpressed cells. We found that GAS6 knockdown decreased the phosphorylation level of Axl in parental cells; however, p-Axl expression was not blocked in CD73 overexpressed cell lines (*SI Appendix*, Fig. S3*D*). We also confirmed these findings in CD73 knockdown cell line with exogenous human recombinant GAS6 stimulation. Western blotting revealed increased protein levels of p-Axl, while the total protein remained unchanged. Moreover, CD73 knockdown blocked GAS6-induced Axl activation compared to control cells (*SI Appendix*, Fig. S3*E*). transwell assays showed that the number of migratory cells was less in the CD73-silenced group than in the control group under the GAS6-treated condition (*SI Appendix*, Fig. S3 *F* and *G*). These findings indicate that CD73 can induce Axl activation independent of GAS6.

### Soluble CD73 can Bind to Axl and Thus Induce Axl Activation.

**Fig. 3.**
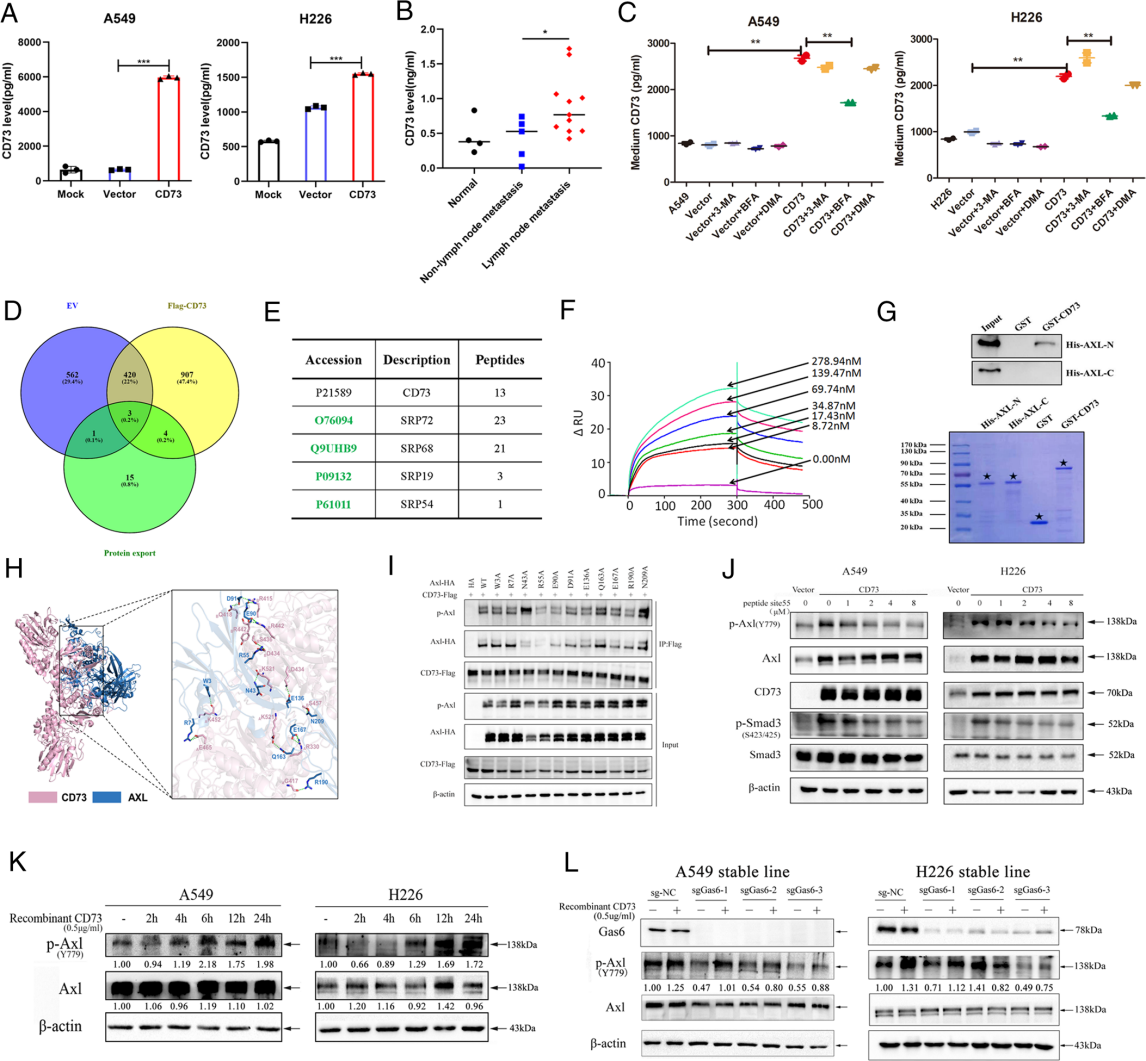
Soluble CD73 can bind to Axl and induce Axl activation. (*A*) ELISA of the basal CD73 secretion level in CD73 overexpressed cell lines and control cells. (*B*) ELISA analysis of the soluble CD73 expression in serum of normal and NSCLC patients with or without lymph node metastasis. (*C*) ELISA of the basal secretion level of CD73 in CD73 overexpressed cell lines and control cells or treated with 50 nM DMA, 1 mM 3-MA, or 10 ng/mL BFA for 24 h. (*D* and *E*) The mass spectrometry assay of the potential CD73-interacting proteins. (*F*) BIAcore assay of Axl activity after the increased concentration of soluble CD73. (*G*) Detection of GST-CD73 bound to His-Axl-N or His-Axl-C by GST pull-down assay. (*H*) The MD simulation of the predicted binding structure and hydrogen bond between CD73 and Axl. (*I*) Co-IP analysis of Axl-HA variants (WT, full-length Axl, W3A, R7A, N43A, R55A, E90A, D91A, E136A, Q163A, E167A, R190A, N209A) with FLAG-CD73 in HEK293T cells using anti-FLAG agarose. (*J*) Western blot assay of p-Axl and p-Smad3 expression in A549 and H226 stable cell lines after treated with peptide site 55. (*K*) Western blot assay of p-Axl and Axl expression in A549 and H226 parental cell lines after stimulated with 0.5 μg/ml recombinant CD73 for 2 h, 4 h, 6 h 12 h, and 24 h individually. (*L*) Western blot assay of p-Axl, Axl, and Gas6 protein levels in Gas6 stable knockout cells after stimulated with 0.5 μg/mL rh-CD73 for 6 h.

The above findings demonstrated that CD73 can promote Axl activation. However, the mechanism remains unclear. Fundamental research has shown that CD73 can function in both cell- and non-cell-bound forms (15). Next, we determined whether soluble CD73 can be secreted then function as a ligand and bind with Axl. First, we examined soluble CD73 in the supernatant of cultured cell lines and found that soluble CD73 was increased after CD73 overexpression in both A549 and H226 cell lines (Fig. 3*A*). Moreover, soluble CD73 was highly expressed in the serum of lung cancer patients with lymph node metastatic status compared with nonmetastatic disease (Fig. 3*B*). CD73 overexpressed cell lines were also treated with the autophagy inhibitor 3-MA, Golgi apparatus transport inhibitor brefeldin A, and exosome biogenesis inhibitor DMA. Only BFA treatment blocked the secretion of CD73, suggesting that CD73 can be secreted via the Golgi apparatus transport pathway (Fig. 3*C*). Then, we performed mass spectrometry analysis in human HEK 293 T cells to determine the potential interacting partners with CD73, and the results showed that signal recognition particle (SRP) may promoted the secretion of CD73 out of the cell (Fig. 3 *D* and *E*).

Next, to investigate whether CD73 directly binds to Axl, we performed the BIAcore assay. And data showed that CD73 directly interacted with Axl, and more importantly, the activation level of Axl increased along with the concentration of soluble CD73 (Fig. 3*F*). Moreover, GST pull-down assays with the extracellular domain of Axl (Axl-N), the intracellular domain of Axl (Axl-C), and CD73 in an *Escherichia coli* system. The purified proteins were identified by SDS-PAGE and western blotting analysis. This assay showed that Axl-N domain directly binds to GST-CD73, but not GST (Fig. 3*G*). These data suggested that the N-terminal extracellular domain of Axl could directly bind to CD73. In addition, the MD simulation method was used to study the interaction and stability of the binding between CD73 and Axl protein. 3D structural models of CD73 and Axl protein are shown in *SI Appendix*, Fig. S4 *A* and *C*. Ramachandran plot data mainly showed the percentage of amino acids located in the fully permissible region (red region), the permissible region (yellow region), and the forbidden region (blank region). We can see that the total amount of the amino acids located in the red region and yellow region exceeded 99%. This finding suggested that the dihedral angles of all amino acid residues of CD73 and Axl protein were within a reasonable range and in accordance with the stereochemical energy rules (*SI Appendix*, Fig. S4 *B* and *D*). ZDOCK data showed that E33-P440 domain of Axl protein binds to the U-shaped cavity formed by the C terminus of CD73 protein dimer (*SI Appendix*, Fig. S4*E*). RMSF and SASA analysis showed the CD73-Axl complex remained stable during the dynamic interaction (*SI Appendix*, Fig. S4 *F* and *G*). Finally, we found that C-terminal domain of B chain of CD73 protein can interact with the N-terminal domain of Axl protein. And the detailed amino acids that form the hydrogen bond between the two proteins are also shown in Fig. 3*H*.

Then, we tried to explore the exact binding site between CD73 and Axl-N extracellular domain. We mutated all the predicted amino acid sites located in Axl and found that mutation of sites N43, R55, E90, D91, E167, and R190 prevent Axl from binding with CD73. However, only mutation of the sites R55, E90, and R190 could reduce Axl phosphorylation (Fig. 3*I*). A peptide is composed of 10 to 15 amino acids, which is a biochemical substance between amino acids and proteins, it is widely used in the study of disease pathogenesis, the development of antibodies and vaccines, and the discovery and development of drugs. To further confirm the exact amino acid sites, we synthesized associated peptides that contained potential Axl binding sites. Then, we treated A549 and H226 overexpression cells with all the peptides. Interestingly, we found that only peptide site 55 significantly blocked the interaction between CD73 and Axl, at the same time, the phosphorylation of SMAD3 can be reduced (Fig. 3*J* and *SI Appendix*, Fig. S5). All above data suggested that CD73 could directly bind with the amino acid site R55 located in Axl extracellular domain and then activate the phosphorylation of Axl. Furthermore, A549 and H226 parental cells were treated with recombinant CD73 and data showed that Axl was initially activated after stimulation in 6 h (Fig. 3*K*). In addition, we constructed GAS6 knockout cell lines using the CRISPR system. We found that recombinant CD73 can activate Axl even under GAS6 knockout conditions, implying that soluble CD73 can function as a ligand for Axl activation independent of GAS6 (Fig. 3*L*).

### CD73 can Stabilize Axl Expression via Inhibiting E3 Ubiquitin Ligases CBLB Expression.

Data from Fig. 2 showed that CD73 can up-regulate both Axl and p-Axl expression. The above data have clarified that soluble CD73 can function as a ligand for Axl activation. How CD73 up-regulates total Axl expression still remained unclear. First, we found that the Axl mRNA expression remained unchanged after CD73 overexpression and knockdown (*SI Appendix*, Fig. S6*A*). And the mRNA expression of known transcriptional factor SP3, YAP1, and APQ of Axl were also unchanged (*SI Appendix*, Fig. S6*B*). Then, we determined whether CD73 can affect Axl expression at the protein level. A549 and H226 CD73-overexpressed cells were treated with CHX to inhibit protein synthesis. We found that the overexpression of CD73 in cells prolonged the half-life of Axl protein (*SI Appendix*, Fig. S6 *C* and *D*). IP experiments confirmed that Axl can bind with HA-labeled ubiquitin and proteasome inhibitor MG132 can further increase its ubiquitination level (*SI Appendix*, Fig. S6*E*). To provide evidence for the role of CD73 in Axl ubiquitination, we cotransfected HEK293T cells with HA-labeled ubiquitin, Flag-labeled CD73 and treated with MG132. Then the cell extract was immunoprecipitated with anti-Axl antibody and detected by immunoblotting with anti-HA antibody. We found that overexpression of CD73 reduced the ubiquitination of Axl (*SI Appendix*, Fig. S6*F*).

Next, we further explored the E3-ligase enzyme that CD73 mediated Axl ubiquitination. Recent published studies showed that Axl was identified as ubiquitylation substrates for E3-ligase CBLB in chronic myeloid leukemia (26). And CBLB also regulates Axl protein stability expressed in natural killer cells to participate in cancer metastasis (27). So we further verified whether CD73 regulates AXL protein expression by affecting CBLB in NSCLC. As shown in *SI Appendix*, Figs. S5 *G* and *H* and S6 *G* and *H*, the CD73 overexpression decreased the mRNA and protein level of CBLB while knockdown of CD73 increased the mRNA and protein level of CBLB. Meanwhile, the expression of Axl was partially restored after transfection of siRNA-CBLB in stable CD73 knockdown cell lines (*SI Appendix*, Fig. S6*I*). These results further confirmed that CD73 can stabilize Axl expression via inhibiting E3 ubiquitin ligases CBLB expression.

### CD73/Axl Promoted NSCLC Cell Metastasis via the Smad3 Signaling Pathway.

**Fig. 4.**
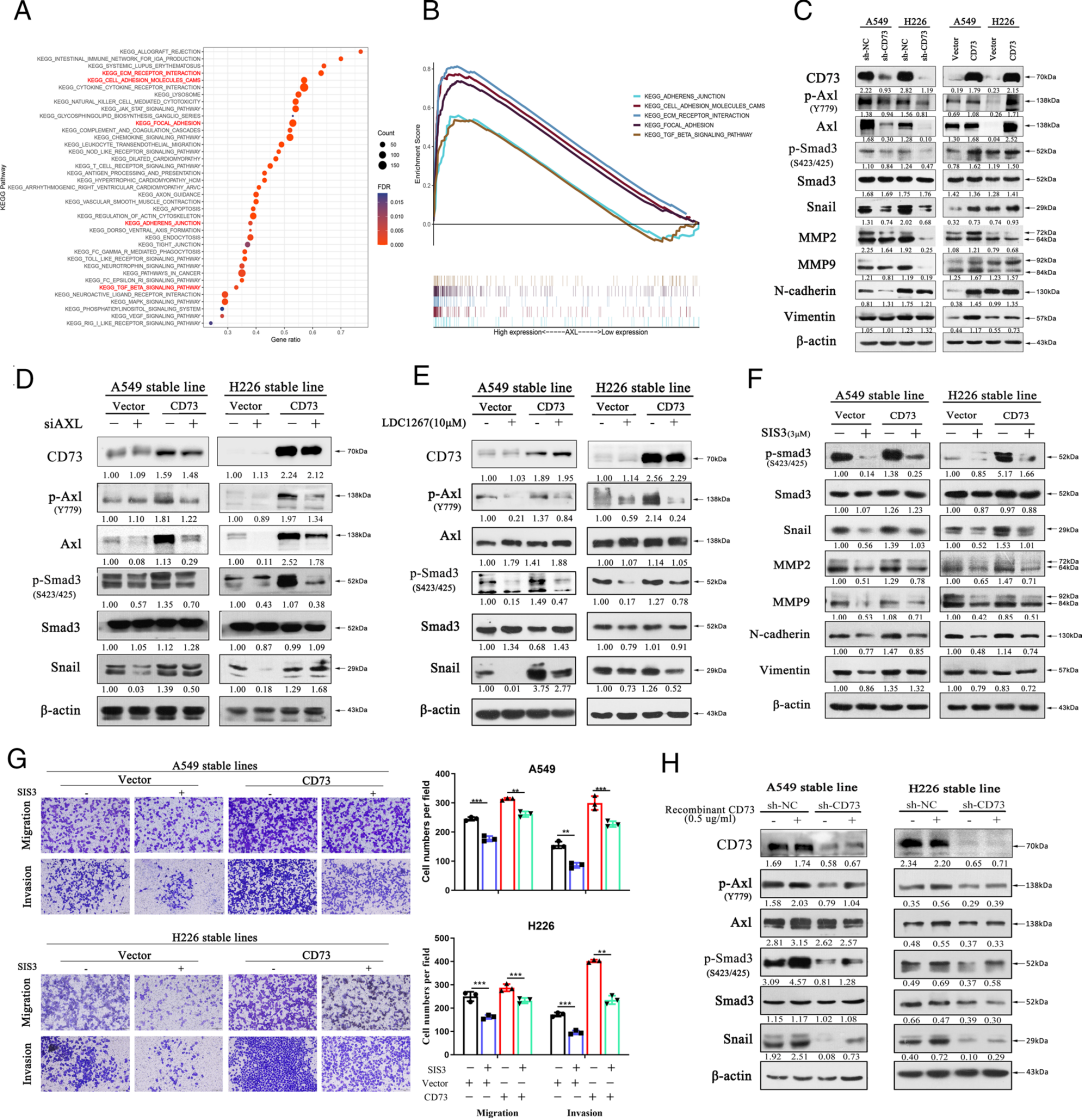
CD73 promotes NSCLC cell metastasis via Smad signaling. (*A* and *B*) The KEGG pathway of Axl interacted proteins. (*C*) Western blot assay of CD73, p-Axl, Axl, p-Smad3, Smad3, Snail, MMP2, MMP9, N-cadherin and Vimentin expression in stable CD73 knockdown or overexpressed cells when compared to control cells. (*D*) Western blot analysis of p-Axl, Axl, p-Smad3, Smad3, and Snail expression in CD73 overexpressed cells after knockdown with si-Axl. (*E*) CD73 overexpressed and control cells were treated with 10 μM LDC1267 for 48 h, then the cell lysates were subjected to western blotting to test p-Axl, Axl, p-Smad3, Smad3, and Snail protein levels. (*F*) CD73 overexpressed and control cells were treated with 3 μM SIS3 for 48 h, then the cell lysates were subjected to western blotting to test p-Smad3, Smad3, Snail, MMP2, MMP9, N-cadherin and Vimentin protein levels. (*G*) The transwell assay of CD73-overexpressed cells and control cells after treated with 3 μM SIS3. (*H*) Western blot assay of p-Axl, Axl, p-smad3, Smad3, and Snail protein levels in CD73 stable knockdown cells after stimulated with 0.5 μg/mL rh-CD73 for 6 h.

Then, we further explored the mechanism beyond CD73/Axl-mediated cell metastasis. KEGG pathway analysis showed that Axl correlated with the TGF-β pathway (Fig. 4 *A* and *B*). Western blot revealed downregulation of p-Smad3, Snail, MMP2, MMP9, N-cadherin, and Vimentin after CD73 knockdown. In contrast, CD73 overexpression showed opposite results (Fig. 4*C*). Then, we knocked down Axl expression with specific siRNA or TAM receptor inhibitor LDC1267 in CD73 overexpressed cells. And we found decreased Axl, p-Axl, and downstream p-Smad3 and Snail protein levels after Axl knockdown. This decrease was weaker in the CD73-overexpressed group (Fig. 4 *D* and *E*). Similar results were obtained in H1299 CD73 overexpressed cells (*SI Appendix*, Figs. S7 *B* and *C* and S8 *D* and *E*). To further verify whether Smad3 signaling participates in CD73/Axl-mediated cell metastasis, CD73-overexpressed cells were treated with Smad3 inhibitor SIS3. And data showed decreased p-Smad3 expression, and downstream snail, MMP2, MMP9, N-cadherin, and Vimentin expression. CD73 overexpression rescued the decreased expression levels of p-Smad3 and downstream protein (Fig. 4*F*), which was in accordance with the transwell data (Fig. 4*G*). Finally, CD73-knockdown and control cells were treated with recombinant CD73, and western blot analysis showed that recombinant CD73 can induce Axl activation and downstream Smad3 signaling. Correspondingly CD73 knockdown blocked rh-CD73-induced up-regulated expression of p-Axl, p-Smad3, and Snail (Fig. 4*H*). Taken together, CD73 can function as a ligand for Axl activation and then activate downstream Smad3 signaling to mediate NSCLC cell metastasis.

### Differential Role of CD73 in Mediating NSCLC Metastasis In Vitro and In Vivo.

It is known that CD73 has both enzymatic and nonenzymatic function and its role is cell type- and tissue-specific (28). Our previous study indicated that CD73 promotes cell proliferation and metastasis in lung squamous cell carcinoma (LUSC) independent of enzymatic activity. To further clarify the distinct enzymatic role of CD73 in mediating lung adenocarcinoma (LUAD) and LUSC, we first examined the expression levels of CD73, Axl, and A2AR in NSCLC cell lines. A2AR was expressed in both LUAD and LUSC cell lines (*SI Appendix*, Figs. S7*A* and S8*A*). We then treated the parental cells with adenosine inhibitor APCP, adenosine analog NECA, and A2AR inhibitor SCH58261. Interestingly, the transwell assay showed that APCP or SCH58261 inhibition or NECA stimulation could regulate cell migration in LUAD A549 and H1299 cell lines. However, these phenomena were not observed in LUSC H226 and SK-MES-1 cells (*SI Appendix*, Figs. S7*A* and S8*A*). NECA was used to stimulate CD73 enzymatic activity. Western blot assay showed that NECA stimulation can induce Axl and downstream Smad3 activation in A549 and H1299 cells; however, no significant p-Axl and p-Smad3 expression was observed in SCC cells (H226 and SK-MES-1) after NECA activation (*SI Appendix*, Figs. S7*B* and S8*B*). In contrary, we observed that APCP and SCH58261 treatment blocked Axl and Smad3 activation in A549 and H1299 cells, but not in H226 and SK-MES-1 cells (*SI Appendix*, Figs. S7 *C* and *D* and S8 *C* and *D*). Next, we also proved similar findings in lung adenocarcinoma CD73-overexpressed cells and CD73-knockdown cells. However, we did not observe similar changes in H226-CD73 overexpressed or knockdown cells (*SI Appendix*, Figs. S7 *E*–*G* and S8 *B, C*, and *E*–*J*). The above findings indicate that CD73 promotes LUAD cell metastasis via CD73 enzymatic activity. This enzymatic role is not essential in LUSC.

**Fig. 5.**
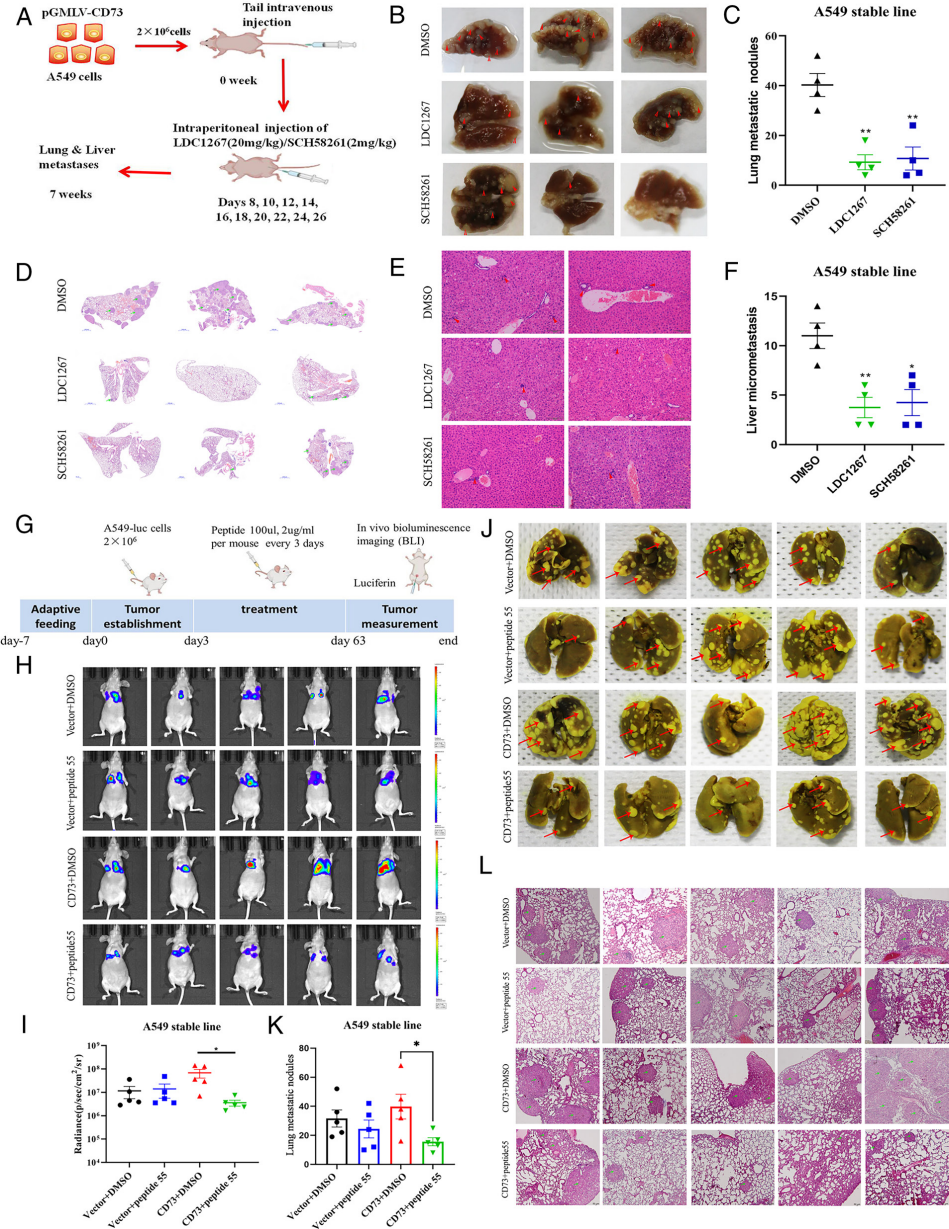
The role of CD73 in mediating lung adenocarcinoma cell metastasis in vivo. (*A*) Graphic diagram of the tumor metastatic model. (*B*) Representative images of surface pulmonary metastasis nodules after treated with LDC1267, SCH58361 in A549 murine model. (*C*) Numbers of lung metastatic nodules in the above treatment groups. (*D*) H&E assay of pulmonary tissues in these groups. (*E*) H&E assay of liver tissues in these groups. (*F*) Numbers of liver micrometastases in the above treatment groups. (*G*) Schematic diagram of in vivo peptide inhibition experiments. (*H*) Representative tumor bioluminescence images of the murine model after treated with peptide. (*I*) The quantification of whole-body luminescence of nude mice in each group after peptide treatment. (*J*) Representative images of surface pulmonary metastasis nodules in murine model. (*K*) The number of lung metastatic nodules in each group. (*L*) H&E assay of lung metastatic nodules in each group.

Then, we verified our findings in vivo. CD73 overexpressed A549 or H226 cells were injected into the veins of nude mice. In the A549-CD73 cell group, we noted that LDC1267 or SCH58261 could decrease the number of metastatic pulmonary nodules (Fig. 5 *A*–*D*) and liver metastasis (Fig. 5 *E* and *F*). Unfortunately, it is very difficult to establish H226-derived tumor metastasis model. We did not observe decreased metastases in lung tissues in the H226 group after LDC1267 or SCH58261 treatment (*SI Appendix*, Fig. S9*A*).

### Synthesized Peptides 55 can Inhibit Cancer Cell Metastasis Mediated by CD73 In Vivo.

The above data showed that CD73 could directly bind with the amino acid site R55 located in Axl extracellular domain and then activate the phosphorylation of Axl. To further assess the effects of the peptide R55 in vivo, we injected A549 stable line cells into the tail vein of nude mice. And then these mice were treated with peptide R55 or DMSO separately. The results showed that the administration of peptide R55 could decrease the lung metastatic nodules in the CD73 overexpressed group, whereas there was no significant difference in vector group (Fig. 5 *G*–*L*). The IHC assay found that the site R55 peptide significantly reduced p-Axl in the overexpressed CD73 group but not in the control groups. (*SI Appendix*, Fig. S9*B*).

**Fig. 6.**
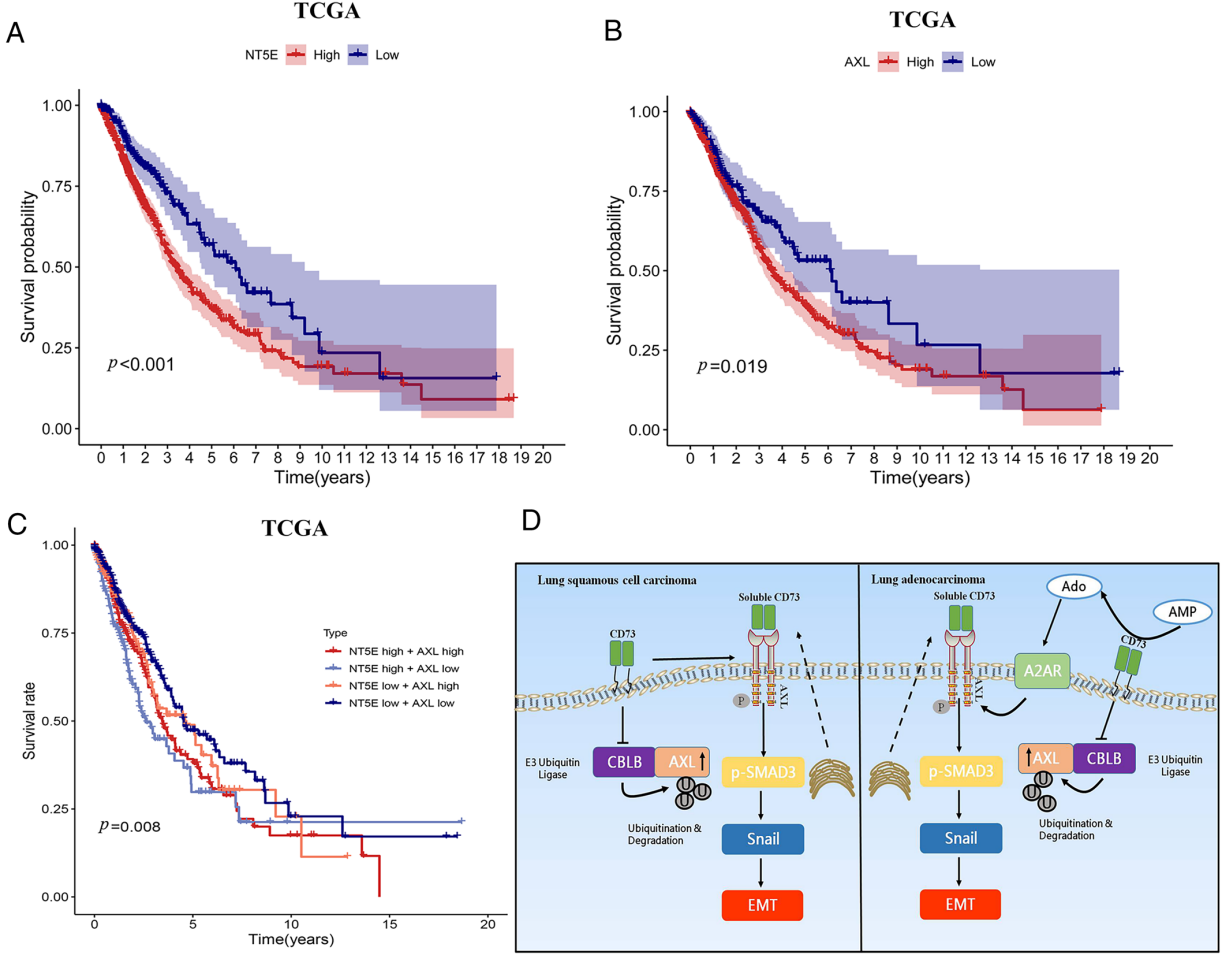
The association between CD73 or Axl expression and patient survival. (*A*) The survival curves of CD73^high^ and CD73^low^ expression and patient survival. (*B*) The association between Axl^high^ and Axl^low^ expression and patient survival. (*C*) The association between CD73^high^Axl^high^, CD73^high^Axl^low^, CD73^low^Axl^high^, CD73^low^Axl^low^ expression, and patient survival. (*D*) The proposed mechanistic model underlying CD73-mediated Axl signaling stimulation in promoting NSCLC cell metastasis under enzymatic or nonenzymatic role. One-way analysis followed by Bonferroni’s post hoc test.

Finally, we verified the data in clinical TCGA database. We found that higher CD73 and Axl expression levels were all correlated with poorer patient survival (Fig. 6 *A* and *B*). And CD73^high^Axl^high^ patients displayed even poorer survival (Fig. 6*C*). Taken together, targeting Axl may be a potential therapeutic strategy in cancer patients with high expression of CD73.

## Discussion

Tumor metastasis is the dominant cause of mortality in NSCLC patients (29). Therefore, identification of metastasis-promoting molecules is important for cancer management and research. In our current study, we found that CD73 was highly expressed in NSCLC tissues and cell lines, which was positively related to the expression of Axl. And overexpression of CD73 can promote NSCLC cell metastasis via the Axl/p-smad3 signaling pathway. Moreover, we found the CD73 can be secreted in soluble form and then directly bind with site R55 located in Axl extracellular domain to serve as a ligand for Axl to induce its activation. In addition, unlike the role in the squamous cell, CD73 can also regulate lung adenocarcinoma cell metastasis via the adenosine-mediated pathway. The above interaction between CD73 and Axl signaling suggested that the combinational analysis of these two biomarkers could predict the prognosis of NSCLC patients more accurately.

Recently, accumulating studies have shown that targeting CD73 is a promising approach in the treatment of various tumors including specific antibody and small-molecule antitumor drugs targeting CD73 (30). In lung cancer, multiple research targets within the adenosine signaling pathway have also been tested in clinical trials (31). Numerous chemicals like BMS-986179, MEDI9447, PT119, and TJ004309 have been used in combination with anti-PD-1/PD-L1 treatment in advanced NSCLC including EGFR mut NSCLC (32). These combinational treatments have made remarkable achievements in improving patient survival. However, the role of CD73 in lung cancer development has not been described totally. And the distinct role of CD73 in LUAD and SCC cell subtype has not been totally demonstrated.

The role of CD73 in mediating tumor cell metastasis has been widely studied (33, 34). In nasopharyngeal carcinoma, CD73 overexpression was related to lymph node metastasis and promoted cell metastasis phenotype (35). In pancreatic cancer, CD73 promoted the metastasis of PDAC by binding to the E3 ligase TRIM21, competing with the Snail for its binding site (36). Our previous study has shown that CD73 knockdown inhibited cell migration and invasion. However, mechanistic evidence for the crucial functions of CD73 as a driver of NSCLC cell metastasis remained largely unknown.

Axl receptor tyrosine is considered as an important player in the context of tumor and immune diseases (37). A well-known activation pathway of Axl is GAS6 ligation (38). In addition, Axl can also trigger self-dimerization or heterodimerization with other RTK family members. Src family kinases were found to bind with Axl via its juxtamembrane domain to trigger ligand-independent autophosphorylation (39). In our study, we surprisingly found that CD73 can directly bind to Axl and induce its activation. Therefore, we hypothesized whether CD73 could function as a ligand for Axl activation.

Soluble CD73 is highly expressed in cancer serum and exosomes and promotes cancer progression (40). Serum CD73 expression can be regarded as an immunological determinant of colorectal liver metastases (41). It is a prognostic factor for metastatic melanoma patients and is associated with response to anti-PD-1 therapy (18). In our study, we innovatively proved that CD73 can be secreted via the Golgi apparatus transport inhibitor pathway. Then it can directly bind with site R55 located in Axl extracellular domain independent of GAS6.

In addition to the phosphorylation levels, the total Axl protein level was also changed, whereas the mRNA level remained unchanged, suggesting that CD73 might regulate Axl expression at the posttranscriptional level. Interestingly, we found that Axl protein can be degraded gradually and CD73 overexpression can reduce the ubiquitination of Axl. CBLB can regulate Axl protein stability in chronic myeloid leukemia and NK cells (26, 27). Similarly, we also proved that CD73 can stabilize Axl expression via inhibiting E3 ubiquitin ligases CBLB expression. Herein, we conclude that CD73 promotes NSCLC cell metastasis via Axl signaling independent of GAS6.

Considering the significant role of CD73-derived adenosine in tumor metastasis, we mainly focused on the enzymatic and nonenzymatic roles of CD73 (42–44). In glioblastoma, CD73 stimulates tumor pathogenesis and enhances cell chemoresistance via the A2B adenosine receptor (45). However, another report showed that in human cervical cancer, CD73 promotes cell proliferation and migration independent of its enzymatic activity (46). Interestingly, our data showed that blockade of the adenosine pathway significantly inhibited cell metastasis and Axl/Smad3 signal pathway activation in LUAD cells. However, we did not observe similar changes in LUSC, suggesting that CD73 enzymatic activity is distinct among subtypes of lung cancer. CD73-promoted LUAD cell metastasis is dependent on enzymatic activity, whereas the nonenzymatic role is dominant in LUSC metastasis.

Our study also demonstrated that SCH58261 or LDC1267 treatment in vivo can inhibit metastasis, but no synergistic inhibitory effect was observed (data not shown). We also confirmed that competitive peptide can significantly reduce the number of lung metastases caused by overexpression of CD73 in vivo. However, this study also has some limitations. We failed to demonstrate the dominant nonenzymatic role in H226 cells in vivo and fewer metastatic nodes were observed in the H226 model, which needs to be verified in future work.

Taken together, our study found that CD73 promotes NSCLC cell metastasis in vitro and in vivo. CD73 expression is positively correlated with Axl expression in cell lines and tissue samples. In addition, this study shows that CD73 can be secreted to serve as a ligand to bind directly to Axl and induce activation in lung cancer. The CD73/Axl interaction can trigger EMT to mediate NSCLC cell metastasis. Moreover, we noted that CD73-promoted lung adenocarcinoma cell metastasis is dependent on enzymatic activity, whereas the nonenzymatic role is dominant in LUSC (Fig. 6*D*). These findings suggest that CD73 targeting should be considered an appropriate candidate for lung cancer therapy.

## Materials and Methods

### Patient Samples.

46 paired NSCLC tissue samples were collected from patients of the First Affiliated Hospital of Soochow University between 2017 and 2020. All participants provided written informed consent at the time of recruitment. All cases had clinically and pathologically confirmed diagnoses of NSCLC in accordance with the Revised International System for Staging Lung Cancer. No cases had received any other treatment before tissue sampling. All collected samples were stored in −80 °C (number/ID of the permission: 2017-164).

### Cell Culture and Drug Treatment.

The cell lines used in this study and culture conditions are described in *SI Appendix*, *Materials and Methods*.

### Establishment of Cell Lines with Stable CD73 Silencing or Overexpression.

CD73-silenced stable cell lines were constructed as previously described (14). The establishment of overexpression CD73 cell line is described in *SI Appendix*, *Materials and Methods*.

### RNA Extraction and Quantitative Real-Time PCR Analysis.

RNA isolation, cDNA synthesis, and quantitative reverse transcription-PCR analyses were performed as previously described (47). The primer sequences used in our study are listed in *SI Appendix*, *Materials and Methods*. The △△Ct method was used to calculate the relative expression levels of these mRNAs.

### Kinase, Western Blot, and Coimmunoprecipitation Assay.

For the human receptor tyrosine kinase (RTKs) assay, we used the phosphorylation antibody array-AAHPRTK-1 (RayBiotech Inc.). Protein lysates were incubated with the array membrane and protein signals were visualized using a chemifluorescence detection system (Bio-Rad) according to the manufacturer’s protocol. The relative density of specific protein expression was determined using Quantity One software. Western blotting and coimmunoprecipitation were performed as previously described (47). The detailed antibodies used in the analysis are listed in supplementary materials. For coimmunoprecipitation assay, IgG-, Axl-, CD73-, HA-, or Flag-bound proteins were isolated via SDS-PAGE and then subjected to western blotting assay.

### Wound Healing, Migration, and Invasion Assays.

The cell wound healing, migration, and invasion assessment was carried out based on the previous studies (14). For the invasion assay, the inserts were precoated with Matrigel matrix (BD Biosciences) diluted in serum-free medium and incubated at 37 °C for 2 h. Finally, the cells were photographed and counted.

### Cell Transfection.

For siRNA or plasmid transient transfection, cells were seeded in 6-well plates until they reached 40 to 60% confluence and then transfected with the expression vectors or specific siRNAs targeting Axl or GAS6 using Lipofectamine 2000 (Invitrogen). The sequence of si-Axl was 5′-ACAGCGAGAUUUAUGACUATT-3′ and si-GAS6 was 5′-GCCUCCAGAUCUGCCACAATT-3′. The detailed methods of construction of CD73 overexpressed plasmids and Gas6 knock-out cells via CRISPR/Cas9 system are shown in *SI Appendix*, *Materials and Methods*.

### ELISA.

The ELISA is described in *SI Appendix*, *Materials and Methods*. CD73 level was measured using a human CD73 ELISA kit (ELH-5NTE, RayBiotech). Gas6 level was measured with a human Gas6 ELISA kit (SEA204Hu, USCN Life Sciences, Wuhan, China).

### GST Pull-Down Assay.

GST-CD73 protein were purified. Then, 5 μg GST-vector or GST-CD73 proteins were added into cell lysates, followed by incubation with Glutathione-Sepharose 4B beads overnight at 4 °C. After removal of nonspecific binding proteins, beads were eluted with 2 × SDS loading buffer. Proteins were examined by SDS-PAGE and western blot analysis.

### Mass Spectrometry Assay.

The affinity-purified samples were analyzed with the Orbitrap Elite hybrid mass spectrometer (Thermo Fisher) coupled with a Dionex LC according to the instructions. Briefly, the cell lysates from HEK 293 T cells transfected with human pCDH-CD73-Flag plasmid and the control were excised and processed for MS analysis. The sample treatment and mass spectrometry assessment were carried out based on the previous studies (47).

### Surface Plasmon Resonance.

Direct binding of human Axl Fc Chimera (R&D, Cat:154-AL), to human CD73 (novoprotein, Cat:C446) was measured by surface plasmon resonance (SPR) with a Biacore T200 instrument. 20 μg/ml of CD73 in 10 mM sodium acetate buffer, pH 4.5 was immobilized on the surface of a Biacore CM5 chip. Purified recombinant human Axl Fc Chimera at different concentrations were infused over the chip at a flow rate of 30 μl/min for 5 min with a running buffer of 10 mM HEPES, 150 mM NaCl, 1 mM MnCl2, 0.05% Tween 20. The dissociation constant was analyzed with Biacore T200 evaluation software.

### Molecular Dynamics (MD) Simulation.

To investigate the potential binding sites between CD73 and Axl RNA, molecular docking and molecular dynamics simulation were carried out. The initial structure of CD73 and Axl were generated. The detailed methods are shown in *SI Appendix*, *Materials and Methods*. Top docking score (1509.65) was selected for further analysis.

MD stimulation of the Axl-CD73 complex was carried out using Gromacs 2018.4 package, applying the Amber14SB all-atom force field combining the TIP3P water model. All hydrogen-related bonds were constrained by the LINCS algorithm. Long-range electrostatics were calculated using the particle–mesh Ewald method. For van der Waals interactions, a cutoff value of 10 Å was used. The configurations were energy minimized and subjected to 1 ns NVT equilibration at 298.15 K. Then, the systems were run for 50 ns of NPT production respectively. The trajectories yielded a total of 5,000 snapshots for production analysis.

### Tumor Metastasis Model.

4 to 6 wk old female BALB/c athymic nude mice were bred under pathogen-free conditions. All animal experiments were performed in accordance with the Soochow University Guide for the Care and Use of Experimental Animals. The control and CD73 overexpressed cells were used to construct the metastatic model. 7 wk after tail vein injection, all mice were killed, then lungs and livers were extracted and fixed with 4% paraformaldehyde. All tissues were subjected to hematoxylin and eosin (H&E) staining.

To test the inhibition efficiency of the peptides in vivo, 2 million stable CD73 cells (A549 Vector/CD73-luc) were subcutaneously injected into the nude mice. 3 d later, the above mice were individually injected with peptide-site55 or DMSO (100μL, 2 μg/mL per mouse) into the tail vein every 3 d. 2 mo later, all mice were set for bioluminescence imaging and then killed for further tissue fixing and H&E staining.

### Bioinformatic Analysis.

NT5E and Axl expression data were downloaded from TCGA database (https://portal.gdc.cancer.gov/). A strong positive correlation was observed between NT5E and Axl in lung adenocarcinoma and lung squamous carcinoma. Single-cell sequencing obtained from the CancerSEA database (http://biocc.hrbmu.edu.cn/CancerSEA/goSearch) showed that both NT5E and Axl expression levels were positively correlated with EMT and metastasis. The GeneMANIA database (http://www.genemania.org) indicated that NT5E, Axl, and GAS6 interact with other genes.

### Statistical Analysis.

All data are presented as mean ± SD. *P* < 0.05 was set as statistically significant (*P < 0.05;**P < 0.01; ***P < 0.001). Data were analyzed by Student’s t test. All statistical analyses were performed using GraphPad Prism (version 6.0; GraphPad, San Diego, CA) and SPSS software (version 17.0; SPSS, Chicago, IL).

## Supplementary Material

Appendix 01 (PDF)

Dataset S01 (XLSX)

## Data Availability

All study data are included in the article and/or *SI Appendix*.
