# Supplementary material for: CD73 promotes non–small cell lung cancer metastasis by regulating Axl signaling independent of GAS6
Source: Proc Natl Acad Sci U S A. 2024 Oct 18;121(43):e2404709121. doi: 10.1073/pnas.2404709121 (PMC11513981; doi:10.1073/pnas.2404709121)
Supplement: Supplementary file 1 — Appendix 01 (PDF) [file pnas.2404709121.sapp.pdf]

## Materials and Methods

### Cell culture and drug treatment

Human bronchial epithelial cells (BEAS-2B) and the human NSCLC cell lines A549, H1299, H1650, PC-9, HCC827, H1975 (lung adenocarcinoma cell line), H226, SK-MES-1, H520, H1703 (lung squamous cell carcinoma) and H460 (human large cell lung cancer) and human embryonic kidney (HEK) 293T cells were obtained from the Cell Bank of the Chinese Academy of Sciences (Shanghai, China). SK-MES-1 cells were cultured in MEM (Gibco, South America, SA, USA), HEK 293T cells were cultured in DMEM (Corning, NY, USA), and all other cells were maintained in RPMI-1640 medium (Corning) supplemented with 10% fetal bovine serum (Gibco) and antibiotics (Invitrogen, CA, USA) at 37 °C in a humidified incubator containing 5% CO2. For inhibitor treatment, control cells and CD73-overexpressed cells were treated with 10 μM LDC1267 (S7638, Selleck), 25 μM SCH58261 (S8104, Selleck), or 10 μM adenosine 5-(α, β-methylene) diphosphate (APCP, M3763, Sigma) for 24 h. The CD73-knockdown cells and control cells were treated with 1 μM 5-(N-ethylcarboxamido) adenosine (NECA, E2387, Sigma) for 24 h. Then, the cells were subjected to a transwell assay. The lysates were analyzed by western blotting.

### Establishment of cell lines with stable CD73 overexpression

To establish cell lines in which CD73 was stably overexpressed, we subcloned the coding sequence of CD73 into the pGMLV-CMV-MCS vector using EcoRI and BamHI endonucleases for expression via a Lenti-X lentiviral expression system (Clontech, Mountain View, CA, USA). The CD73 expression construct was co-transfected with packaging plasmids into HEK293T cells using the PEI reagent. The empty vector served as a negative control. After incubation, the packaged lentiviruses were collected and used to infect the A549 and H226 cells. After 2 days, stable cells were selected using 2 μg/mL puromycin (Beyotime, Shanghai, China).

### Construction of plasmids

The coding sequences of CD73 (GenBank Accession number: NM_001146312.2) were amplified using the corresponding primers (forward: 5′-AATATAGCTAGCATGTGTCCCCG AGCCGC-3′; reverse: 5′-GGCGGGATCCCTATTGGTATAAAACAAAG-3′), followed by subcloning into a pCDH-flag vector to generate pCDH-CD73-Flag. Axl (NM_005901.5) was

amplified with the corresponding primers (forward: 5′-GTAGCTAGCATGGCGTGGCG GTGCCCCAG-3′; reverse: 5′-ATTTGCGGCCGCTCAGGCACCATCCTCCTGCCC-3′) and subcloned into a pcDNA3.1 vector to generate pcDNA3.1-Axl-HA. Before transient transfection, the Flag-tagged CD73 or HA-tagged Axl sequences of the plasmid constructs were confirmed by direct sequencing. The Axl mutants were generated using a Mut Express II Fast Mutagenesis Kit V2 (C214-01, Vazyme). The Axl truncations (1-451, and 452-894 aa) and mutations were generated by mutagenesis PCR.

**CRISPR/Cas9 construct and lentivirus production**

Lenti-Guide CRISPR vector (Genomeditech, Shanghai, China) was used to create the lentivirus CRISPR constructs. Briefly, single guide RNA (sgRNA) sequences (sgRNA1, 5′-CCGCCGGCTGCTCACCGTCT-3′; sgRNA2, 5′-GTTCGAGAACGACCCCGAGA-3; sgRNA3′, 5′-AACTGCGTGGCCTCGCGCGC-3′) targeting GAS6 were designed, synthesized, and annealed. Annealed sgRNA oligonucleotides were ligated into a lenti-guide CRISPR vector digested with Esp3I (BsmBI). The incorporated sgRNA was verified by sequencing each construct. Each lenti-guide CRISPR construct was co-transfected with a packaging plasmid mix (Genomeditech, Shanghai, China) into HEK293T cells. After transfection for 48 h, HEK293T medium containing the lentivirus was harvested and used to infect A549 and H226 cells. Stable A549 and H226 cells were selected using 2 μg/mL puromycin (Beyotime) and confirmed by western blotting.

**Primer sequences of quantitative real-time PCR analysis**

RNA isolation, cDNA synthesis, and quantitative reverse transcription-PCR analyses were performed as previously described [47]. The primer sequences used for CD73 mRNA detection were 5′-TCTTCTAAACAGCAGCATTCC-3′ (forward) and 5′-CATTTCATCCGT GTGTCTCAG-3′ (reverse). For Axl, 5′-CGTAACCTCCACCTGGTCTC-3′ (forward) and 5′-TCCCATCGTCTGACAGCA-3′ (reverse) were used. For Gas-6, 5′-GGACATGGACACCTGTGAGG-3′ (forward) and 5′-GGCCCAGGTACAAGGACTTC-3′ (reverse) were used. β-actin served as an internal control. β-actin mRNA was detected using 5'-CACAGAGCCTCGCCTTTGCC-3' (forward primer) and 5'-ACCCATGCCCACC ATCACG-3' (reverse primer). The △△Ct method was used to calculate the relative expression levels of these mRNAs.

**Western blot, and co-immunoprecipitation assay**

The detailed antibodies used in the analysis were anti-CD73 (13160), anti-Axl (8661), anti-GAS6 (67202), anti-Smad3 (9523), anti-p-Smad3 (9520), anti-Snail (3895), anti-MMP2 (40994), anti-MMP9 (13667), anti-Flag (14793), anti-HA (3724) (Cell Signaling Technology, Danvers, MA, USA), anti-N-cadherin (610920), anti-vimentin (550513) (BD Biosciences), and anti-pAxl (AF2228; R&D Systems). Anti-β-actin and anti-mouse or anti-rabbit secondary antibodies (Cell Signaling Technology) were used. For co-immunoprecipitation assay, protein lysates were incubated with anti-FLAG M2 affinity gel (M8823, Sigma), anti-HA magnetic beads (HY-K0201, MCE).

**ELISA**

A549 and H226 parental cells and A549- and H226-CD73-overexpressing cells were exposed to medium containing 1% FBS for 12 h and then treated with one of the following inhibitors: autophagy inhibitor 3-methyladenine (1 mM, S2767, Selleck), protein transport inhibitor brefeldin A (10 ng/mL, B1400, APExBIO), and exosome secretion inhibitor, 5-(N, N-dimethyl)-amiloride DMA (50 nM, C3505, APExBIO). The supernatant was collected from the culture medium after 24 h. Normal serum samples were taken from non-tumor patients and other serum samples were taken from the previous 46 patients.

**Molecular Dynamics (MD) Simulation**

To investigate the potential binding sites between CD73 and Axl RNA, molecular docking and molecular dynamics simulation were carried out. The initial structure of CD73 was generated based on the Homologous sequence obtained from NCBI database (PDB ID 4H1S) and then the crystal structure of CD73 was further modeled with Swiss-Model program. The 3D structure model of Axl was generated with RoseTTAFold program. Then Axl was docked into the CD73 RNA binding region using ZDOCK 3.2 software. Top docking score (1509.65) was selected for further analysis.

**Tumor metastasis model**

In the metastatic model, the control and CD73 overexpressed cells were suspended in PBS ($2 \times 10^6$ cells/mouse) before injected into mouse via tail vein. To evaluate the effects of inhibitor treatment, the mice were randomly divided into three groups: (1) 5% DMSO control (i.p. every other day); (2) LDC1267 (20 mg/kg, i.p. every other day); (3) SCH58261 (2 mg/kg, i.p.

every other day); (n = 4 per group).

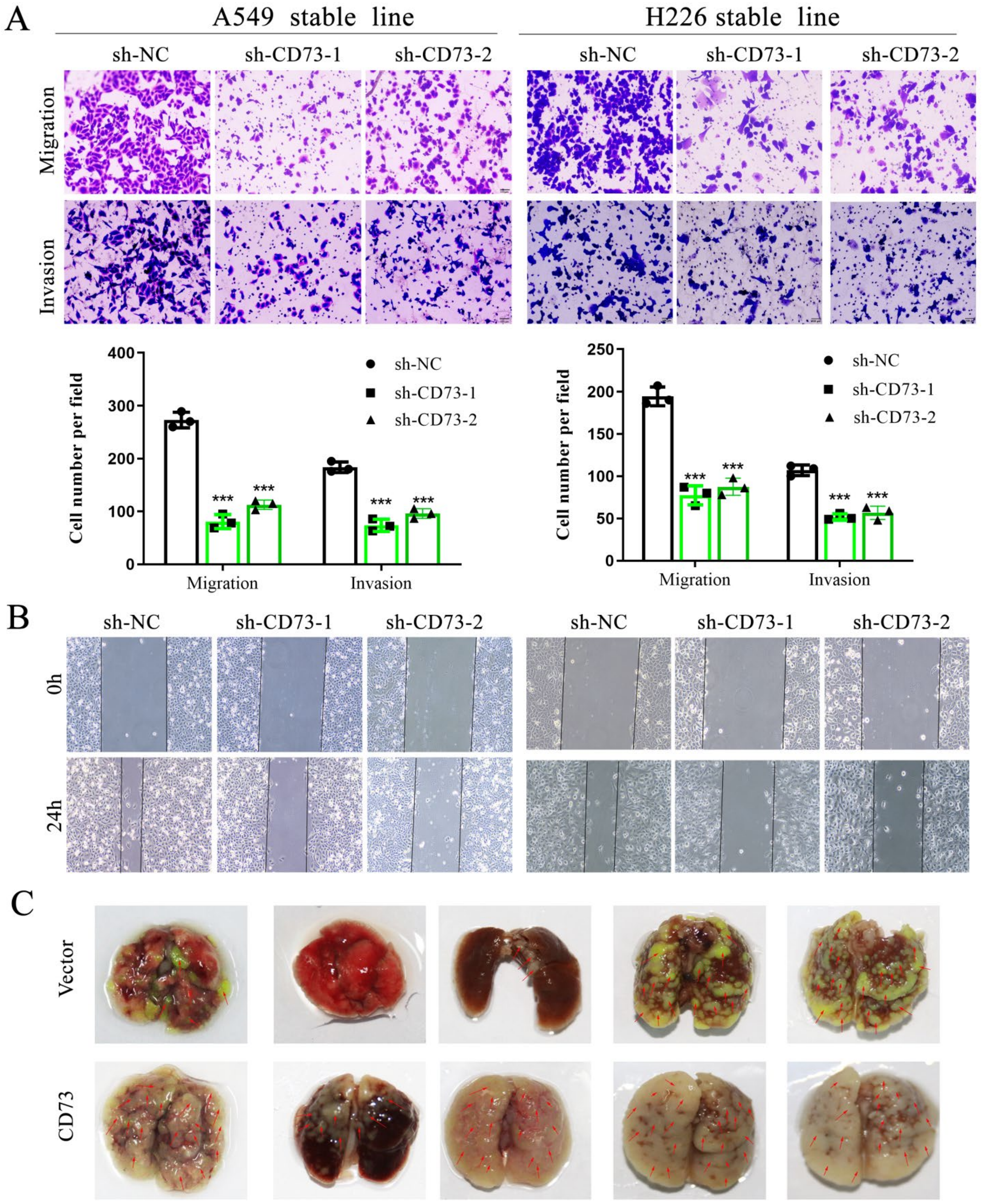


**Figure S1. CD73 regulates NSCLC cell metastasis. (A)** Transwell assay of A549 and H226 cells after CD73 knockdown. (**B)** Wound healing assay of A549 and H226 cells after CD73 knockdown. (**C)** Representative images of surface pulmonary metastasis nodules after CD73 overexpression in murine model. Data were presented as the mean ± SD. Data were analyzed using non-paired Student's t-test. ***P < 0.001 vs. control or as indicated.

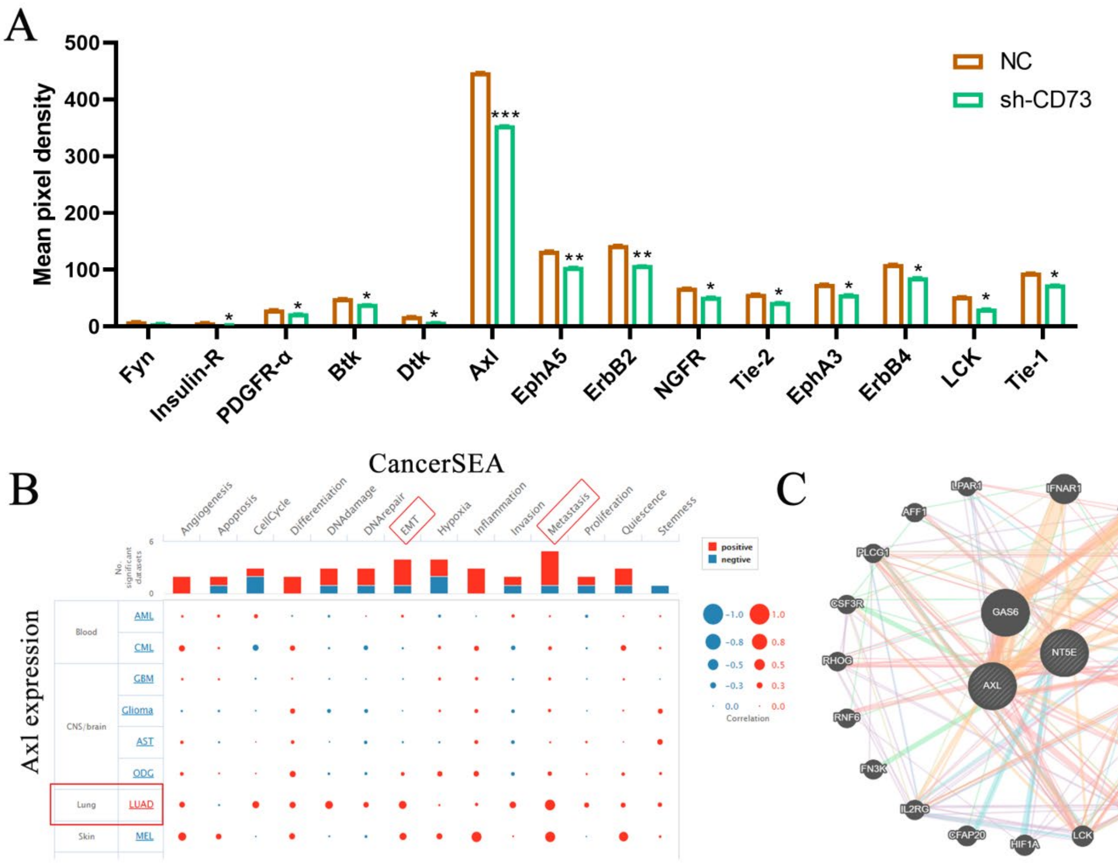


**Figure S2. The interactions between CD73 and AXL, Gas6. (A)**RTK protein assay of down-regulated proteins in sh-NC and sh-CD73 group. **(B)** The association between Axl expression and EMT/metastasis via CancerSEA database. **(C)** The interactions between NT5E, Axl and Gas6 via GeneMANIA database.

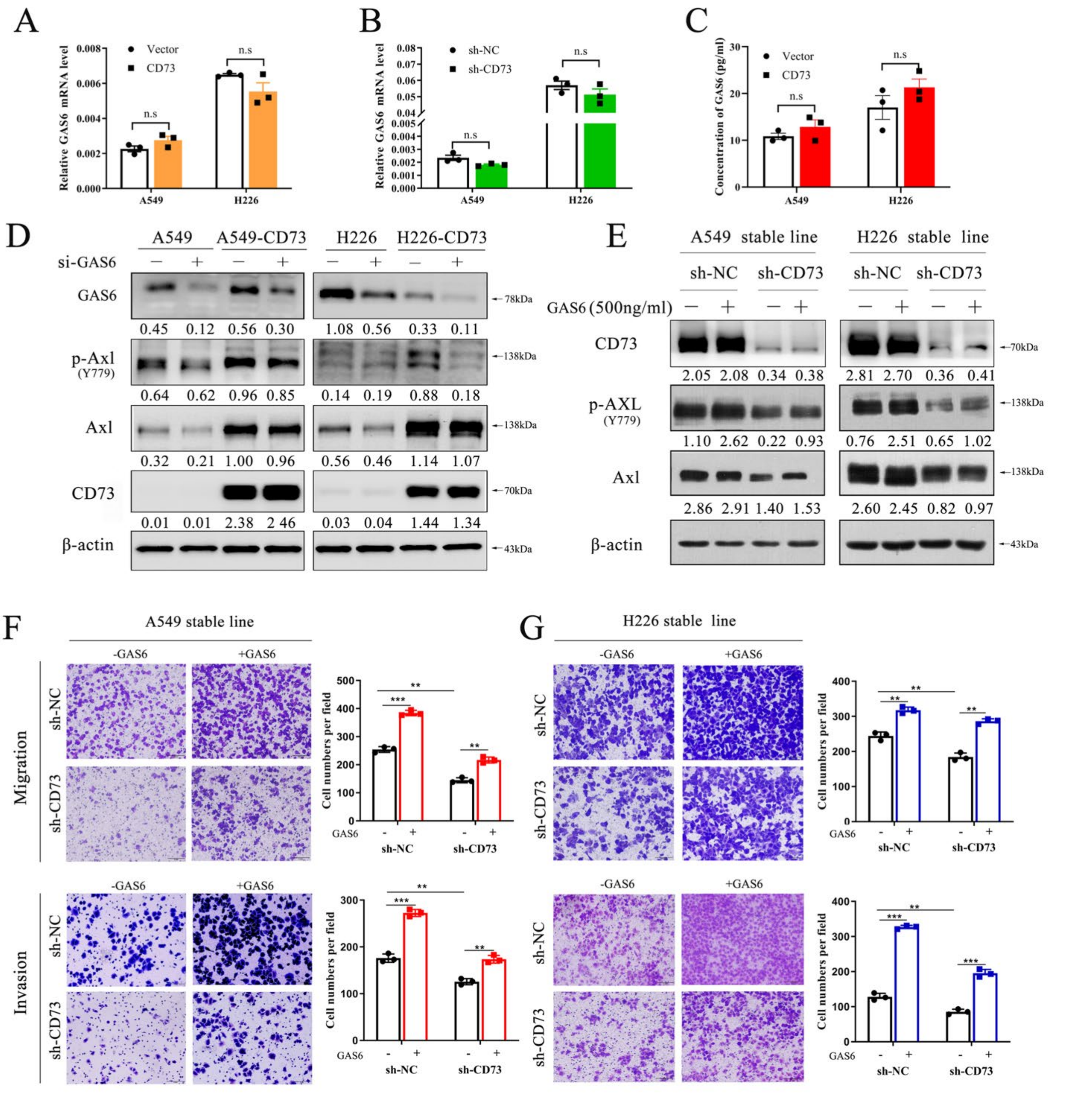


**Figure S3. CD73 induces Axl activation independent of Gas6. (A and B)** qRT-PCR analysis of Gas6 mRNA expression levels in stable CD73 knockdown and overexpression cell lines. (**C)** ELISA of Gas6 basal secretion level in CD73 overexpressed cell lines and control cells. **(D)** Western blot assay of p-Axl and Axl in parental cells and CD73 overexpressed cells after knockdown with si-Gas6 **(E)** Western blot assay of p-Axl, Axl protein levels in CD73 stable knockdown cells after stimulated with 500 ng/mL rh-Gas6 for 6 h. (**F and G)** Transwell assay in CD73-knockdown cells and control cells after stimulated with Gas6.

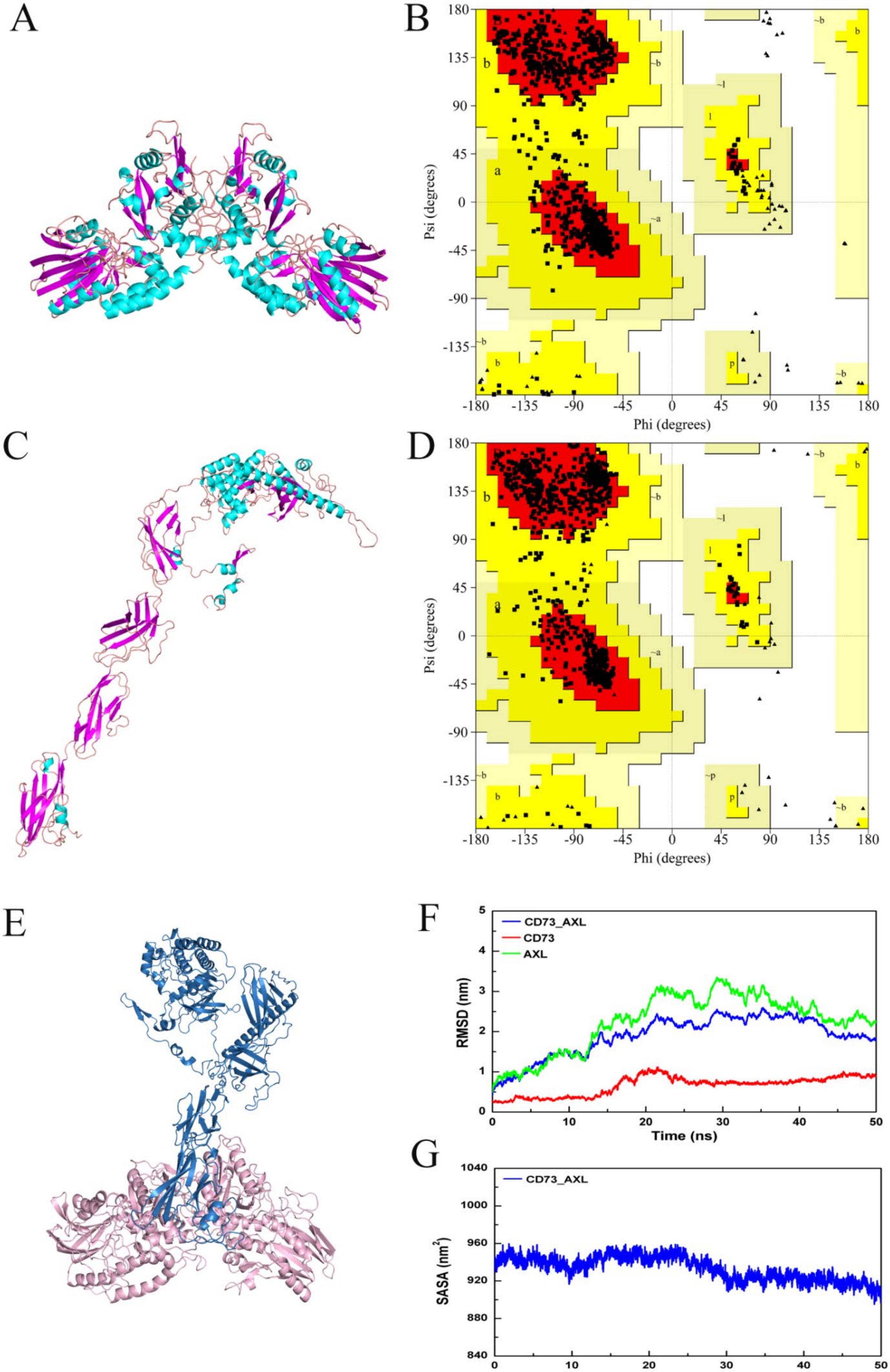
A
B
C
D
E
F
G
Psi (degrees)
Phi (degrees)
CD73_AXL
CD73
AXL
RMSD (nm)
Time (ns)
SASA (nm$^2$)

**Figure S4. The MD simulation of interaction between CD73 and Axl complex. (A, C)** 3D structural models of CD73 protein using SWISS-MODEL procedure and Axl protein using RoseTTAFold procedure. (**B, D)** Ramachandran plot of percentage of the amino acids located in the fully permissible region (red region), the permissible region (yellow region) and the forbidden region (blank region). € The ZDOCK docking results between CD73 and Axl. (**F)** ZDOCK results of the changes of Root mean square deviation (RMSD) values of CD73, Axl monomer and CD73-Axl complex over time. (**G)** The changes of Solvent Accessible Surface Area (SASA)value of CD73-Axl complex over time.

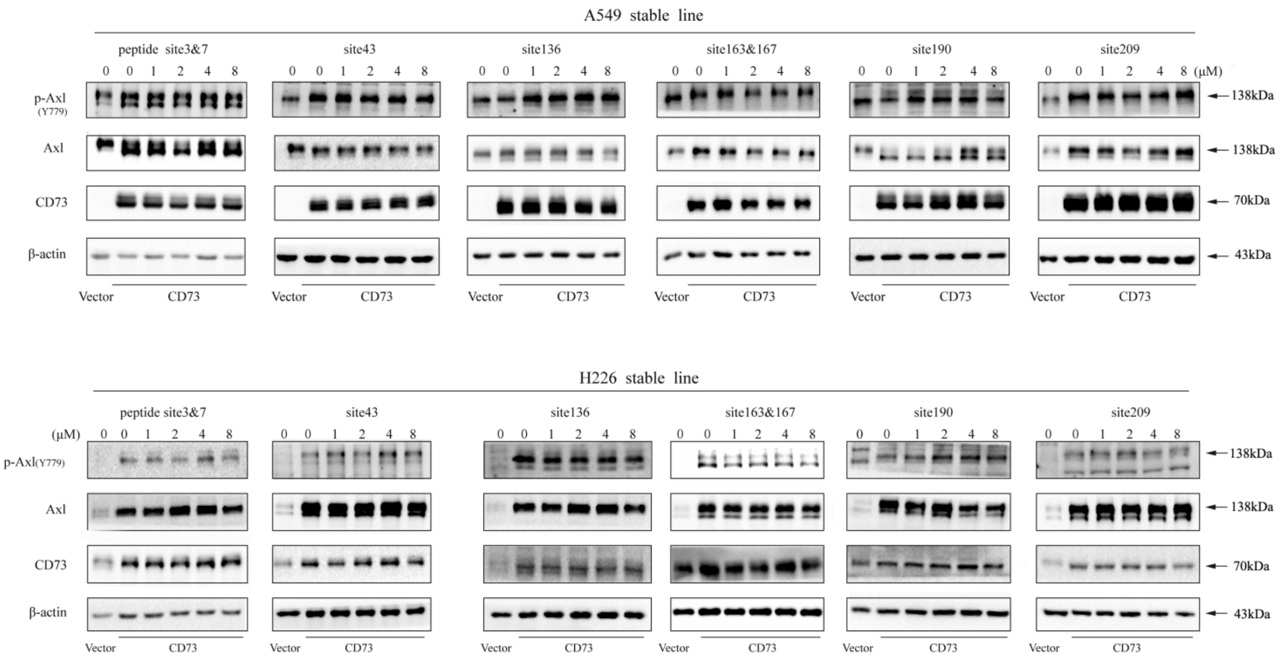


**Figure S5.** Western blot assay of p-Axl and Axl expression in A549 and H226 stable cell lines after treated with different peptides.

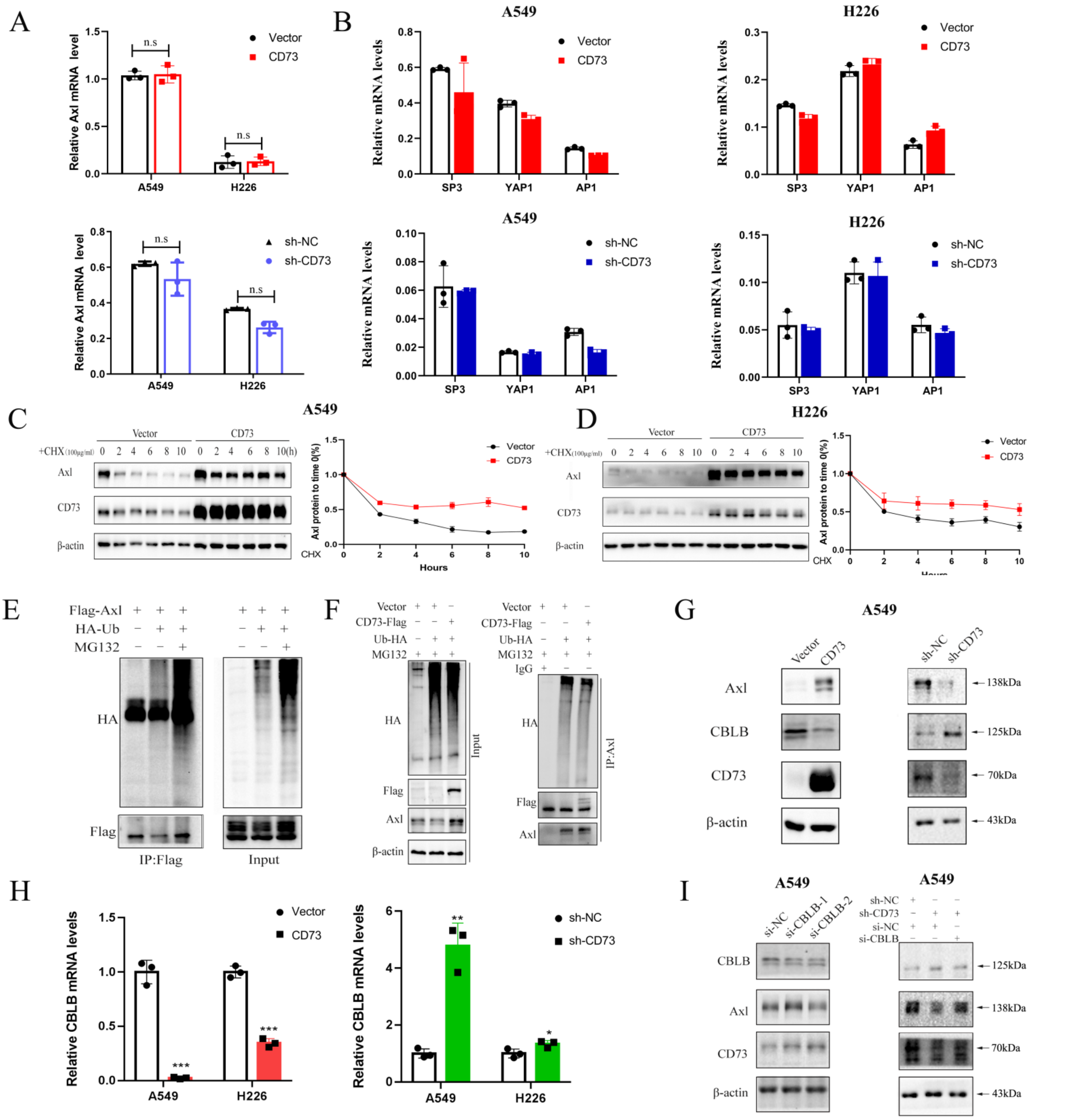

A
B
A549
H226
Vector
CD73
sh-NC
sh-CD73
n.s
Relative Axl mRNA level
Relative mRNA levels
SP3
YAP1
AP1
C
D
+CHX(100μg/ml)
0 2 4 6 8 10(h)
Axl
CD73
β-actin
Axl protein to time 0(%)
Hours
CHX
E
Flag-Axl
HA-Ub
MG132
HA
Flag
IP:Flag
Input
F
CD73-Flag
Ub-HA
IgG
IP:Axl
G
si-NC
si-CBLB-1
si-CBLB-2
CBLB
138kDa
125kDa
70kDa
43kDa
H
Relative CBLB mRNA levels
I
si-CBLB

**Figure S6. CD73 can stabilize Axl expression via inhibiting E3 ubiquitin ligases CBLB expression. (A)** Axl mRNA expression levels in stable CD73 overexpressed and knockdown cell lines via qRT-PCR assays. (**B)** Relative SP3, YAP1 and AP1 mRNA expression in stable CD73 overexpressed and knockdown cell lines via qRT-PCR assays. (**C and D)** Western blot assay of Axl and CD73 expression in A549 and H226 stable CD73 overexpressed cells and control cells after treated with 100 μg/ml CHX for 2 h, 4 h, 6 h, 8 h, 10 h. (**E**) HEK293T cells were transfected with Flag-Axl or HA-Ub plasmids and then treated with MG132 (10μM). (**F)** HEK293T cells were transfected with control or Flag-CD73 and HA-Ub plasmids and treated with MG132 (10μM). (**G)** Western blot assay of CBLB expression in A549 and H226 stable CD73 overexpressed cells. **(H)** Relative CBLB mRNA expression in stable CD73 overexpressed and knockdown cell lines via qRT-PCR assays. (**I)** Western blot assay of Axl expression in A549 cells or CD73 knockdown cells after silenced with si-CBLB.

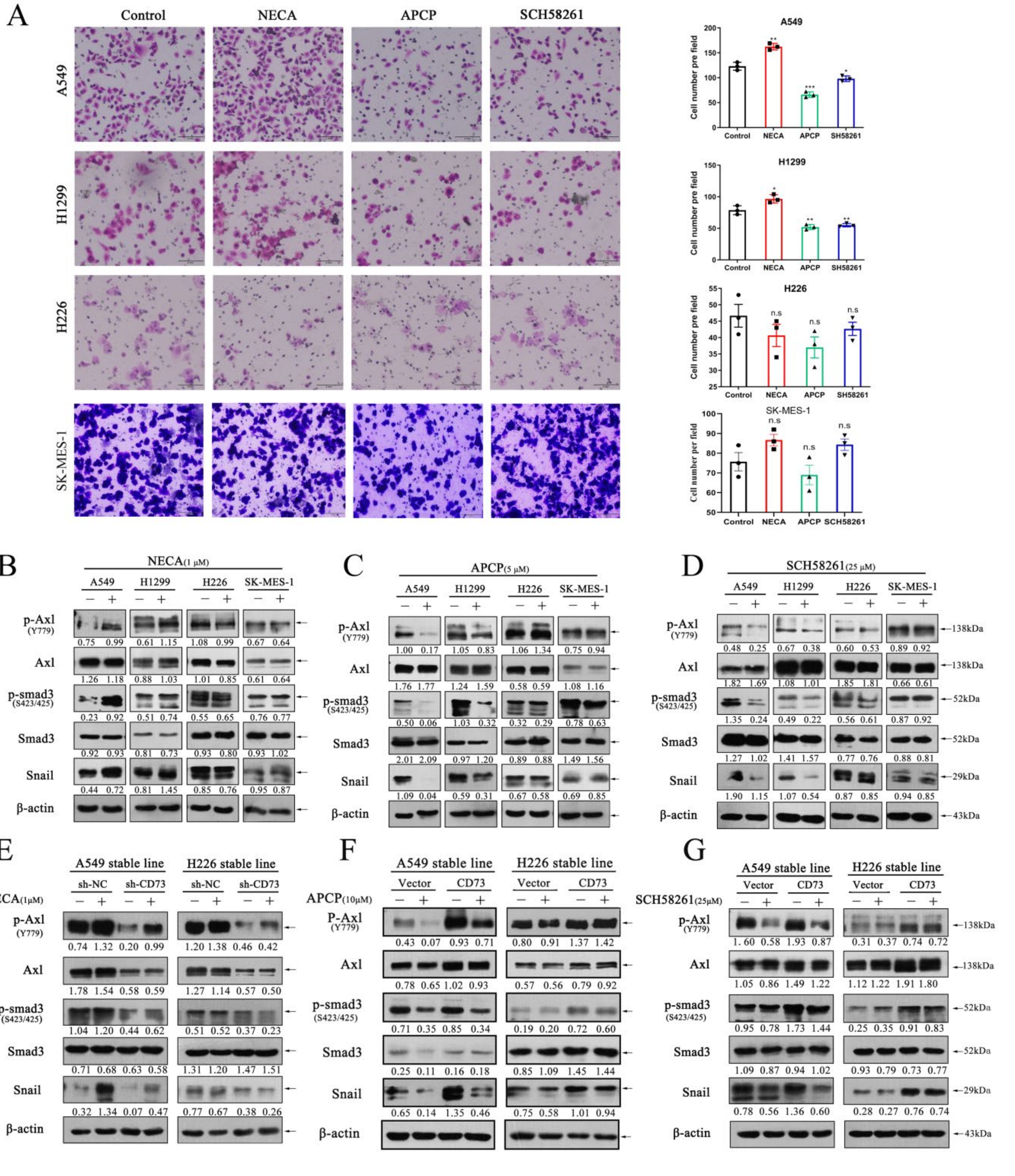
A
Control
NECA
APCP
SCH58261
A549
H1299
H226
SK-MES-1
Cell number pre field
B
NECA(1 μM)
C
APCP(5 μM)
D
SCH58261(25 μM)
p-Axl (Y779)
Axl
p-smad3 (S423/425)
Smad3
Snail
β-actin
E
A549 stable line
H226 stable line
sh-NC
sh-CD73
F
Vector
CD73
G
138kDa
52kDa
29kDa
43kDa

**Figure S7. The enzymatic role of CD73 in mediating NSCLC metastasis in vitro.** (**A)** Transwell assay in A549, H1299, H226 and SK-MES-1 parental cells after treated with 1 μM NECA, 10 μM APCP and 25 μM SCH58261 separately. (**B)** Western blot assay of p-Axl, Axl, p-smad3, Smad3 and Snail protein levels in A549, H1299, H226 and SK-MES-1 parental cells after stimulated with 1 μM NECA for 24 h. (**C and D)** Western blot assay of p-Axl, Axl, p-smad3, Smad3 and Snail protein levels in A549, H1299, H226 and SK-MES-1 parental cells after treated with 10 μM APCP or 25 μM SCH58261 for 48 h. (**E)** Western blot assay of p-Axl, Axl, p-smad3, Smad3 and Snail protein levels in CD73 stable knockdown cells after stimulated with 1 μM NECA for 24 h. (**F and G)** Western blot assay of CD73, p-Axl, Axl, p-smad3, Smad3 and Snail expression in stable CD73 overexpressed cells and control cells after treated with 10 μM APCP or 25 μM SCH58261 for 48 h. One-way analysis followed by Bonferroni's post hoc test. Ns, no significance.

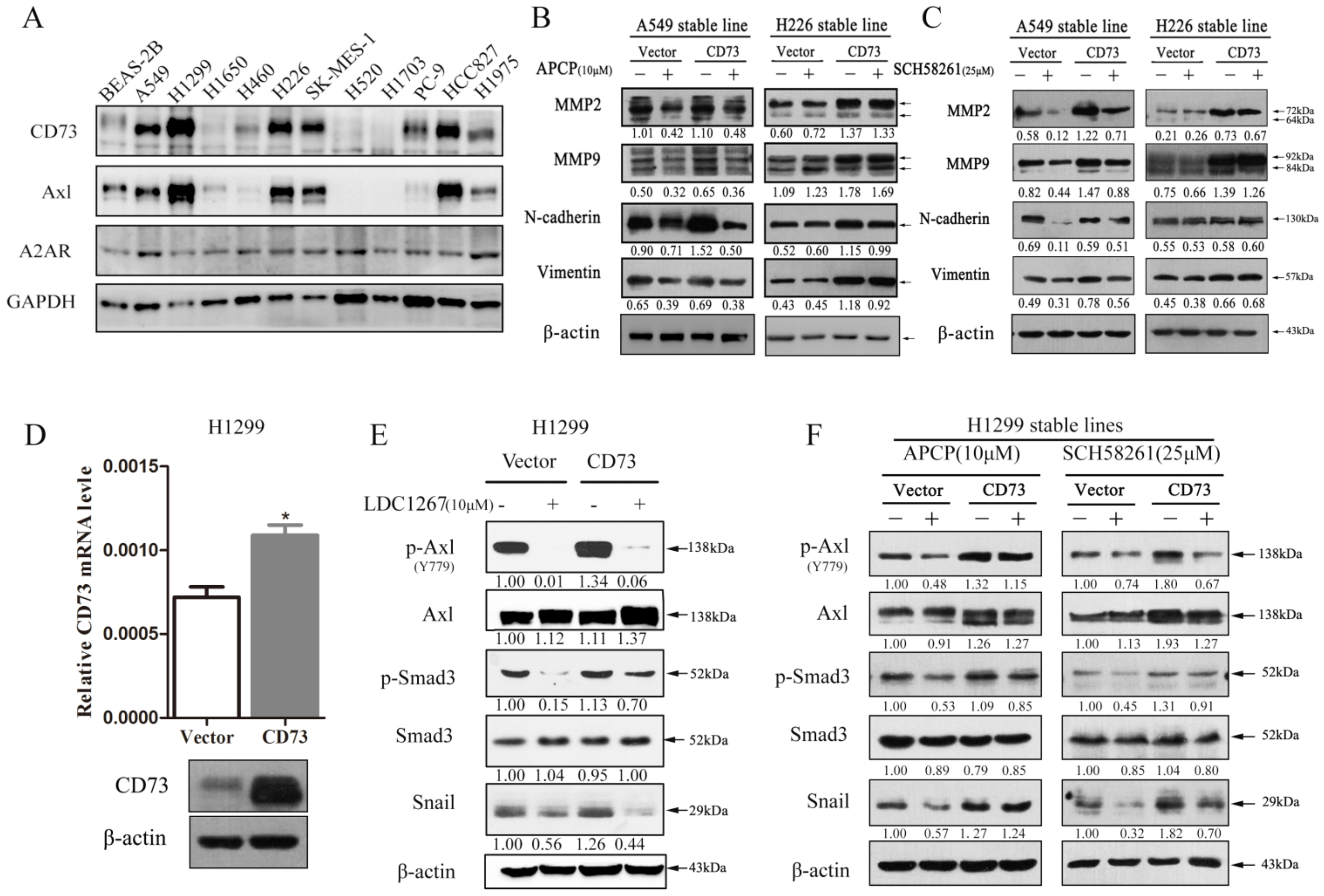

**Figure S8. The enzymatic role of CD73 in mediating NSCLC metastasis in vitro. (A)** Western blot assay of CD73, Axl and A2AR expression in BEAS-2B, A549, H1299, SPC-A1, H1650, H460, Calu-3, 95C, 95D, 16HBE, H226, SK-MES-1, PC-9, H1299 and HCC827 cell lines. **(B and C)** Western blot assay of MMP2, MMP9, N-cadherin and Vimentin expression in stable CD73 overexpressed cells and control cells after treated with 10 μM APCP or 25 μM SCH58261 for 48 h. **(D)** CD73 mRNA and protein expression levels after stable knockdown and overexpression in H1299 cell lines via qRT-PCR and western blot assays. (**E**) The expression levels of p-Axl, Axl, p-Smad3, Smad3, and Snail in H1299-CD73 overexpressed and control cells after treated with 10 μM LDC1267 for 48 h. **(F)** Western blot assay of p-Axl, Axl, p-smad3, Smad3 and Snail expression in H1299-CD73 overexpressed cells and control cells after treated with 10 μM APCP or 25 μM SCH58261 for 48 h.

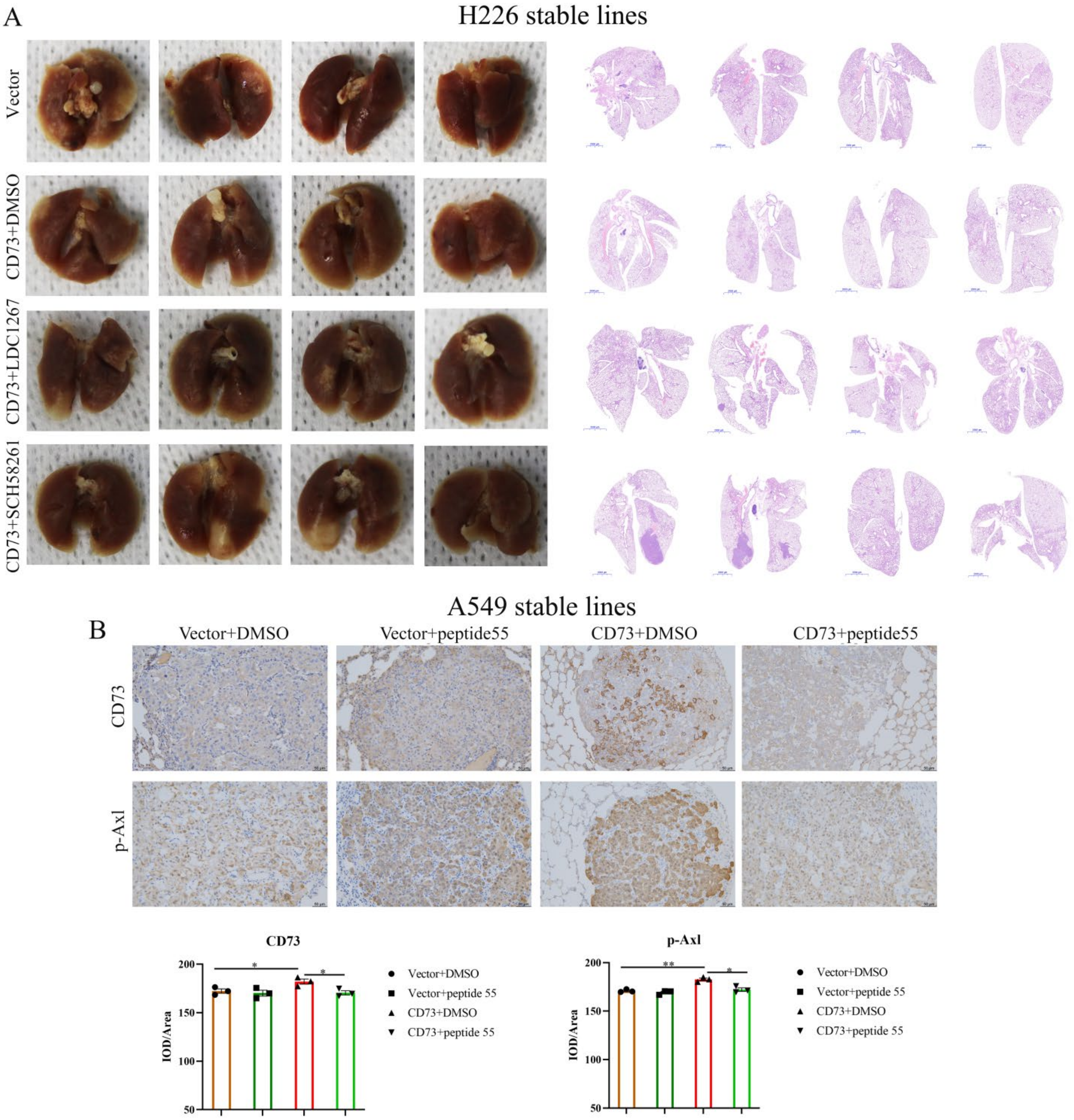


**Figure S9. Representative images in A549 and H226 model. (A)**Representative images of surface pulmonary metastasis nodules and H&E images after treated with LDC1267, SCH58361 in H226 murine model. (B) Representative images of CD73 expression intensity of pulmonary metastasis nodules after treated with peptide 55 in A549 murine model.

**Table S1.** Demographic and clinical characteristics of NSCLC patients and the level of CD73 mRNA expression in tumor tissue specimens

| **Characteristics** | **Number(%)** | **CD73 mRNA expression** | | | |
|---|---|---|---|---|---|
| | | **high** | **low** | **$\chi 2$** | ***p* value** |
| Age | | | | | |
| ≤65 | 19(41.30) | 12 | 7 | 0.002 | 0.968 |
| >65 | 27(58.70) | 16 | 11 | | |
| Gender | | | | | |
| Male | 27(58.70) | 11 | 16 | 0.718 | 0.396 |
| Female | 19(41.30) | 11 | 8 | | |
| Histology | | | | | |
| Adenocarcinoma | 27(58.70) | 18 | 9 | | |
| Squamous cell carcinoma | 14(30.43) | 6 | 8 | 2.159 | 0.339 |
| Others | 5(10.87) | 3 | 2 | | |
| Degree of differentiation | | | | | |
| Low | 13(28.26) | 5 | 8 | 2.621 | 0.105 |
| Middle | 33(71.74) | 23 | 10 | | |
| Smoking status | | | | | |
| Yes | 18(39.13) | 12 | 6 | 0.056 | 0.812 |
| No | 28(60.87) | 19 | 9 | | |
| Clinical stage | | | | | |
| I/II | 29(58.70) | 17 | 12 | 0.088 | 0.767 |
| III/IV | 17(41.30) | 10 | 7 | | |
| Lymph node metastasis | | | | | |
| No | 30(65.21) | 11 | 19 | 4.695 | **0.030** |
| Yes | 16(34.79) | 12 | 4 | | |

P: Chi-Square Test

**Table S2**. Demographic and clinical characteristics of NSCLC patients and the level of CD73 protein expression in tumor tissue specimens

| **Variables** | **Number (%)** | **CD73 protein expression** | | | |
|---|---|---|---|---|---|
| | | **Low** | **High** | $\chi 2$ | ***P*-value** |
| Gender | | | | | |
| Male | 9(45) | 3 | 6 | 0.108 | 0.742 |
| Femal | 11(55) | 4 | 7 | | |
| Age | | | | | |
| ≤ 65 year | 6(30) | 3 | 3 | 0.167 | 0.682 |
| >65 years | 14(70) | 4 | 10 | | |
| Histology | | | | | |
| Squamous carcinoma | 5(25) | 2 | 3 | 0.073 | 0.786 |
| Adenocarinoma | 15(75) | 5 | 10 | | |
| T status | | | | | |
| T1-2 | 14(70) | 4 | 10 | 0.167 | 0.682 |
| T3-4 | 6(30) | 3 | 3 | | |
| N status | | | | | |
| N0 | 13(65) | 3 | 10 | 1.065 | 0.302 |
| N1-3 | 7(35) | 4 | 3 | | |
| Clinical stage | | | | | |
| I | 7(35) | 2 | 5 | | |
| II | 4(20) | 1 | 3 | 0.655 | 0.720 |
| III | 9(45) | 4 | 5 | | |

P: Chi-Square Test
